# Ambient Stable Triboelectric Nanogenerator Based on Conductive Filler Modified Silicone Rubber with Gas Barrier Encapsulation for Footstep Energy Conversion

**DOI:** 10.1002/advs.202519523

**Published:** 2025-12-17

**Authors:** Yi Wei, Yushu Tian, Xiaokang Chen, Junfeng Chu, Junjie Wang, Xiangyu Chen, Wenjie Wu, Liqun Zhang

**Affiliations:** ^1^ State Key Laboratory of Organic‐Inorganic Composites College of Materials Science and Engineering Beijing University of Chemical Technology Beijing 100029 P. R. China; ^2^ Institute of Emergent Elastomers School of Materials Science and Engineering South China University of Technology Guangzhou 510640 P. R. China; ^3^ Beijing Key Laboratory of Micro‐nano Energy and Sensor Beijing Institute of Nanoenergy and Nanosystems Chinese Academy of Sciences Beijing 100083 P. R. China; ^4^ State Key Laboratory of Fluorine and Nitrogen Chemicals School of Chemical Engineering and Technology Xi'an Jiao Tong University Xi'an Shaanxi 710049 P. R. China

**Keywords:** ambient stable, footstep energy conversion, self‐powered motion sensing, triboelectric nanogenerators

## Abstract

Human motion, particularly foot‐ground interaction during locomotion, generates substantial biomechanical energy that remains largely underutilized. Triboelectric nanogenerators (TENGs) have emerged as a promising solution for harvesting such energy, yet their long‐term performance under ambient humidity remains a major challenge for real‐world deployment. Herein, a spring‐assisted contact‐separation mode TENG is reported, composed of high‐temperature vulcanized (HTV) silicone rubber filled with 1 phr of conductive carbon black (600JD). The addition of 600JD increases the surface charge density by 55% compared to unfilled silicone rubber. The optimized device delivers a peak power density of 179.9 mW·m^−2^ and is capable of powering over 1,900 commercial LEDs. To overcome moisture sensitivity, a flame‐retardant chlorinated isobutylene‐isoprene rubber (CIIR) encapsulation layer with excellent gas barrier properties is introduced, enabling stable operation across a broad relative humidity range (30–90% RH). The device retains 98.3% of its initial short‐circuit current after more than 1 000 000 mechanical cycles, indicating exceptional durability. Beyond energy harvesting, the TENG also functions as a self‐powered sensor capable of footstep detection, step frequency monitoring, and motion pattern recognition. This work presents a resilient and scalable design strategy for ambient‐stable TENGs toward footstep energy harvesting and intelligent sensing under variable environmental conditions.

## Graphical abstract

A humidity‐tolerant triboelectric nanogenerator is developed using conductive carbon black‐modified silicone rubber and a flame‐retardant gas barrier layer. The device efficiently harvests footstep energy while maintaining stable output under 30–90% RH and over 1 million cycles. It also functions as a self‐powered motion sensor, offering a scalable strategy for ambient‐stable biomechanical energy conversion.

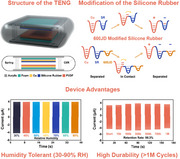

## 1 Introduction

With the growing urgency for sustainable energy development and the rapid expansion of the Internet of Things (IoT), energy harvesting technologies have garnered considerable attention across various scientific and engineering disciplines.^[^ ^1^, ^2^, ^3^, ^4^ ^]^ Among the available ambient sources, high‐entropy mechanical energy, characterized by low density, intermittency, and structural irregularity, poses significant challenges for traditional energy conversion strategies.^[^ ^5^ ^]^ These energy sources include mechanical vibrations,^[^ ^6^, ^7^, ^8^ ^]^ human motion,^[^ ^9^ ^]^ acoustic waves,^[^ ^10^, ^11^, ^12^ ^]^ and wind,^[^ ^13^, ^14^ ^]^ which are often difficult to capture using conventional electromagnetic or piezoelectric devices. To address these limitations, TENGs, first introduced in 2012,^[^ ^15^ ^]^ have emerged as a promising technology for harvesting mechanical energy from diverse, irregular sources.^[^ ^16^, ^17^, ^18^, ^19^ ^]^ Based on the coupling of contact electrification and electrostatic induction,^[^ ^20^ ^]^ TENGs generate electrical output through repeated contact and separation of dielectric layers. Unlike traditional generators, TENGs can efficiently convert low‐frequency, non‐periodic, and small‐amplitude motions into electricity.^[^ ^21^ ^]^ Their lightweight structure, high voltage output, and compatibility with flexible materials make them ideal for powering microelectronic systems, particularly in soft robot,^[^ ^22^ ^]^ wearable^[^ ^23^, ^24^, ^25^, ^26^ ^]^ and IoT‐based^[^ ^27^, ^28^ ^]^ applications, providing an innovative approach for harvesting human kinetic energy.^[^ ^29^ ^,^ ^30^ ^]^

Among the various biomechanical sources, human foot motion is especially attractive due to its high frequency, force intensity, and regularity during everyday activities. Studies estimate that an average adult produces ≈100 W of mechanical power daily, with foot movements alone contributing up to 67 W.^[^ ^31^, ^32^ ^]^ Activities such as walking, running, and climbing stairs consistently generate untapped biomechanical energy, much of which dissipates as heat.^[^ ^33^, ^34^ ^]^ Efficient harvesting of footstep energy not only enables sustainable powering of wearable electronics but also supports the realization of autonomous, self‐powered sensing platforms. Consequently, footstep energy harvesting has become a key research direction in ambient energy utilization.^[^ ^35^ ^]^ Despite recent progress, one of the major bottlenecks hindering the real‐world application of TENGs lies in their vulnerability to environmental humidity, which leads to charge dissipation and performance degradation. Moreover, as demonstrated in recent composite‐based TENGs,^[^ ^36^, ^37^, ^38^, ^39^ ^]^ material‐level optimization using functional fillers or hybrid films plays a critical role in enhancing output and long‐term durability.

In this work, we develop a spring‐assisted contact‐separation TENG optimized for footstep energy harvesting. The device utilizes HTV silicone rubber modified with 1 phr (parts per hundred rubber) of 600JD, which boosts the surface charge density by 55%. Unlike the widely studied nanofillers such as CNTs, AgNWs, and MXene, carbon black, despite its extensive industrial usage, has remained underexplored in triboelectric applications. In this work, we demonstrate that integrating 600JD carbon black into an HTV silicone rubber matrix not only enhances charge density but also retains industrial processability and material flexibility, offering a scalable, low‐cost alternative for durable TENG design. Additionally, a sandpaper‐based surface microstructuring technique is employed to improve triboelectric output. The resulting device achieves a peak power density of 179.9 mW·m^−2^, sufficient to power over 1900 commercial LEDs and charge a 47 µF capacitor to 3 V within 3 min.

**Figure 1:**
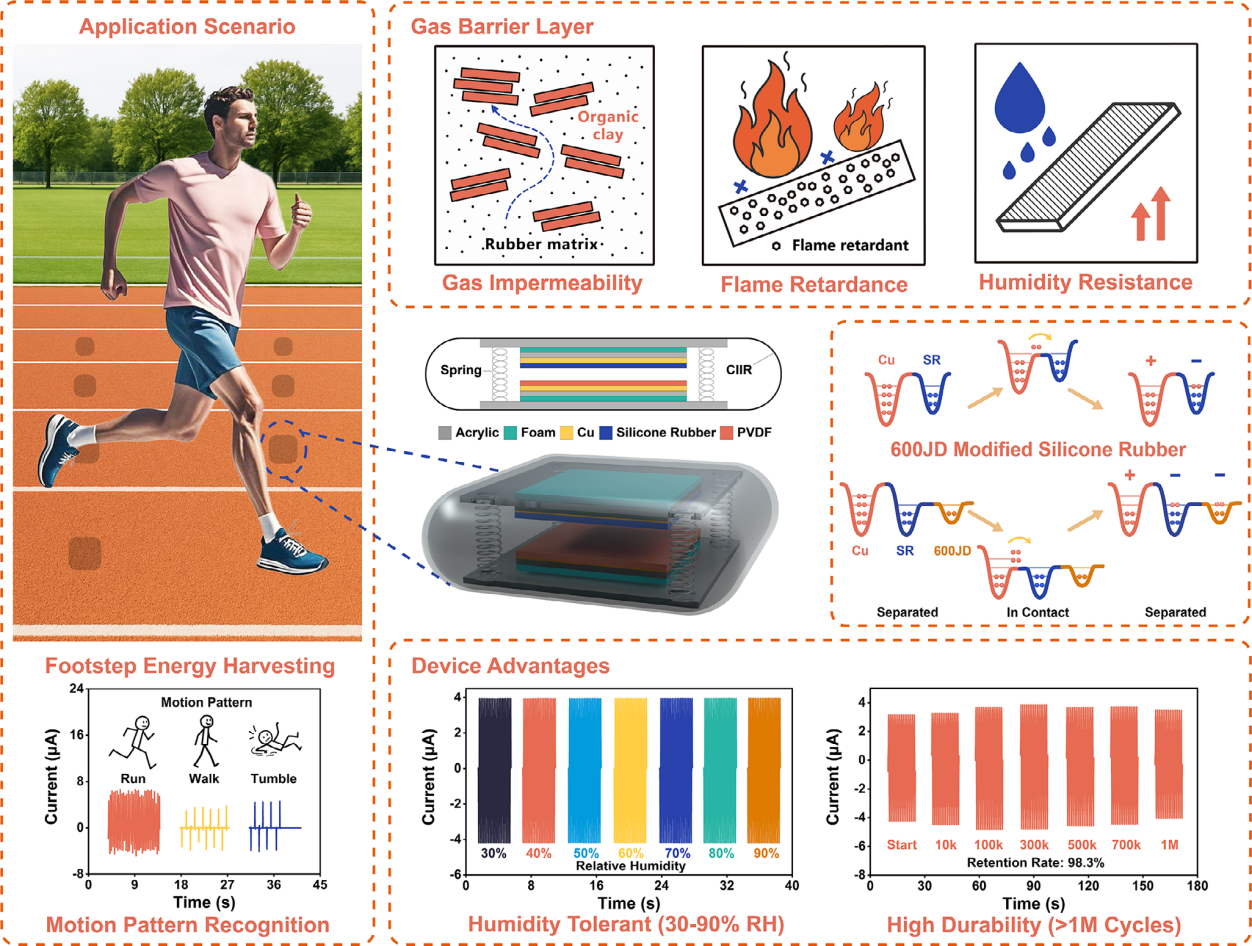
Illustration of the structural design, performance advantages, and potential application scenarios of the TENG.

To ensure stable operation under humid conditions, a flame‐retardant CIIR was selected as a multifunctional encapsulation material that integrates moisture barrier performance, flame retardancy, and mechanical elasticity, offering a rare combination not commonly achieved in previously reported TENG systems. Compared with typical polymeric encapsulants such as PET, PE or PDMS,^[^ ^40^, ^41^, ^42^ ^]^ the flame‐retardant CIIR developed in this work is more suitable for real‐world integration in wearable and motion‐interactive applications due to its superior safety and mechanical adaptability. This modification allows the TENG to maintain electrical output across a wide humidity range (30%–90% RH) and retain 98.3% of its output after more than 1 000 000 mechanical cycles. The encapsulation design not only ensures environmental stability but also maintains mechanical compliance, enabling continuous output under harsh dynamic conditions. Beyond energy harvesting, the device also functions as a self‐powered motion sensor, capable of detecting footstep patterns and monitoring step frequencies. The structural design, performance advantages, and potential application scenarios of the developed TENG are shown in **Figure** **1**. This study presents a durable and scalable TENG design that integrates material and structural innovations to overcome environmental instability. By combining conductive filler modification with gas‐barrier encapsulation, this work provides a practical approach for deploying TENGs in footstep energy harvesting and intelligent motion sensing under real‐world conditions.

## 2 Results and Discussion

### 2.1 Triboelectric Performance of Silicone Rubber Composites with Various Fillers

Silicone rubber is widely adopted in TENGs due to its inherent flexibility, which facilitates intimate contact with counter surfaces and enhances charge transfer during contact electrification.^[^ ^43^, ^44^ ^]^ To further boost its triboelectric output, numerous strategies have been reported, including the incorporation of high‐permittivity fillers ,^[^ ^45^, ^46^ ^]^ chemical functionalization with polar groups,^[^ ^47^, ^48^ ^]^ and the construction of microstructured surfaces.^[^ ^49^, ^50^ ^]^

**Figure 2:**
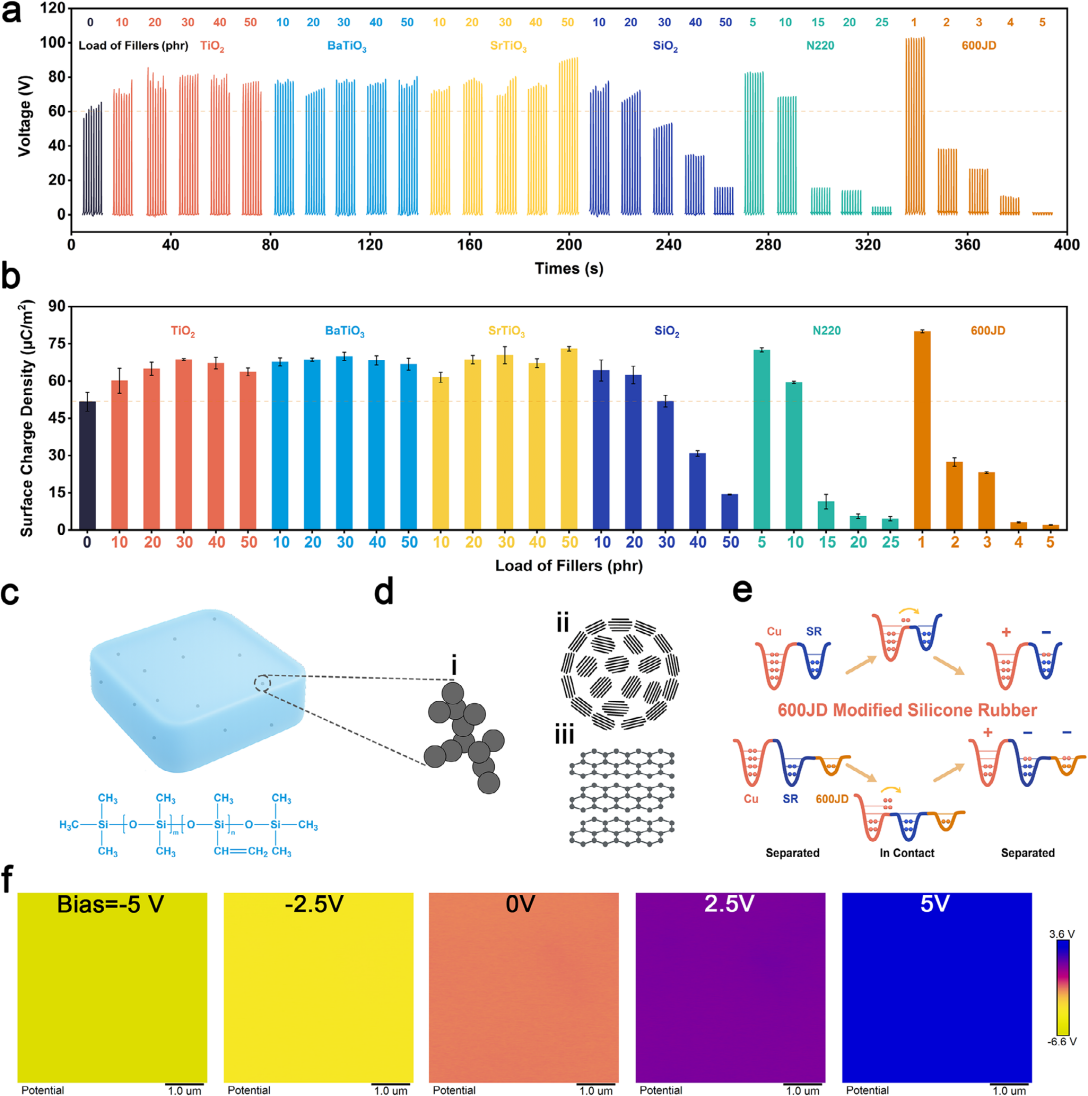
Triboelectric performance of silicone rubber composites with six different fillers. a) Open‐circuit voltage and b) Surface charge density of silicone rubber filled with six different fillers when tested against a copper film. c) Molecular structure of methyl vinyl silicone rubber and schematic illustration of the silicone rubber/600JD composite. d) Schematic illustration of the multiscale microstructure of carbon black: i) carbon black, ii) hierarchical clusters, and iii)nanoscale graphitic domains. e) Schematic diagram of the mechanism by which 600JD enhances the surface charge density of silicone rubber during the contact‐electrification with copper. f) KPFM characterization results of the surface potential of 1 phr 600JD‐filled silicone rubber under different bias voltages. All images share the same scale bar to ensure comparability.

In this study, HTV silicone rubber was selected as the matrix material due to its favorable mechanical properties, enhanced crosslinking reactivity, and low compression set. Its molecular structure is illustrated in **Figure** **2**c. To systematically investigate the effect of filler type on triboelectric performance, six representative fillers were incorporated into the silicone rubber matrix. These included three widely studied dielectric fillers in TENGs (TiO_2_, BaTiO_3_, and SrTiO_3_), two conventional rubber reinforcement fillers (SiO_2_ and industrial‐grade carbon black N220), and one conductive carbon black (600JD), which has been rarely explored in TENG systems but is systematically evaluated here due to its unique electrical characteristics. The fillers were loaded at different concentrations ranging from 10 to 50 phr (for dielectric and reinforcing fillers), and from 1 to 5 phr for 600JD. The composites were prepared via a two‐roll mill followed by a two‐step vulcanization process using hydroxyl silicone oil and bis(2,5‐dimethyl‐2,5‐di‐tert‐butylperoxy)hexane as additives.

The triboelectric performance of the composites was evaluated via contact‐separation tests against a copper electrode. As summarized in Figure 2a,b, TiO_2_, BaTiO_3_, and SrTiO_3_ exhibited a modest increase in surface charge density and open‐circuit voltage at low filler contents, followed by saturation or slight decline at higher concentrations. These trends are consistent with theoretical predictions based on the parallel‐plate capacitor model (Equation 1) .^[^ ^51^ ^]^

(1) $$Q=C\cdot V_{TENG}=\varepsilon_{0}\varepsilon_{r}\cdot \frac{A}{d}\cdot V_{TENG}$$

where Q is the surface charge, C is the capacitance, *V_TENG_* is the triboelectric voltage, *ε_0_* is the vacuum permittivity, *ε_r_* is the relative dielectric constant of the triboelectric material, A is the effective contact area, and d is the thickness of the TENG. This equation indicates that both *ε_r_* and *V_TENG_* play critical roles in determining the total surface charge output. By increasing the dielectric constant through the incorporation of high‐permittivity fillers such as BaTiO_3_, the surface charge density can be significantly improved. Based on this understanding, many studies have attempted to enhance the performance of triboelectric materials by incorporating dielectric fillers. The trends observed with TiO_2_, BaTiO_3_, and SrTiO_3_ in our study align well with these findings.

For SiO_2_‐filled composites, triboelectric output peaked at 10 phr and decreased thereafter. This is attributed to the reinforcing nature of SiO_2_, which stiffens the silicone matrix and reduces surface conformity during contact, thereby lowering effective contact area and charge transfer efficiency. In contrast, both N220 and 600JD showed an initial rise followed by rapid deterioration at higher loadings. These results align with percolation theory, wherein conductive fillers beyond the percolation threshold form continuous conductive networks that facilitate charge leakage, thereby suppressing triboelectric output.

Among all tested fillers, 600JD demonstrated the most significant enhancement. Remarkably, as can be seen in Figure 2b, the surface charge density increased by 55% at only 1 phr loading compared to pristine silicone rubber, outperforming all other filler types. This suggests that minimal 600JD addition can substantially improve TENG performance without compromising insulation. A similar trend was observed in the short‐circuit current output, as shown in Figure S1 (Supporting Information). N220 and 600JD are both carbon blacks, but with distinct morphology and electronic characteristics. As illustrated in Figure 2d, carbon black (i) consists of nanoscale graphitic (iii) domains aggregated into hierarchical clusters(ii). Compared to N220, 600JD possesses a significantly higher specific surface area, contributing to superior conductivity. Based on the electron cloud model for contact electrification, we propose a mechanism to explain how 600JD improves the triboelectric performance of silicone rubber, as shown in Figure 2e. When 600JD is added in small amounts, it stays dispersed in the matrix without forming a conductive network. During contact with copper, the 600JD particles offer extra space for storing transferred electrons, which remain there after separation and lead to higher surface charge density. The surface potential of silicone rubber with 1 phr 600JD is measured using Kelvin probe force microscopy (KPFM), as shown in Figure 2f. The results show that the surface potential increases with the tip bias, which agrees with previous reports. The images share the same scale bar to ensure comparability. Additional KPFM images without normalized color scaling, along with the corresponding AFM topography map, have been provided in Figure S2 (Supporting Information) to better visualize local surface potential variations and their correlation with surface morphology. To understand the influence of filler composition on dielectric behavior, frequency‐dependent permittivity and electrical conductivity were also systematically evaluated for six types of composites. As presented in Figures S3 and S4 (Supporting Information), the incorporation of a small amount of 600JD (1 phr) significantly enhanced the dielectric constant while maintaining low dielectric loss and high volume resistivity, which is favorable for charge accumulation in triboelectric applications. In contrast, excessive loading (>2 phr) led to a percolation transition, resulting in increased leakage and reduced surface charge density. Dielectric fillers, including SiO_2_, TiO_2_, BaTiO_3_, and SrTiO_3_, exhibited insulation stability over the entire concentration range, but their ability to enhance permittivity was relatively limited.

In addition, to further examine whether the conductive carbon black alters the interfacial electronic structure of the silicone matrix, high‐resolution XPS spectra of the C 1s, O 1s, and Si 2p regions were additionally collected for HTV silicone rubber /600JD composites with different filler loadings, as shown in Figure S5 (Supporting Information). These spectra show no observable binding‐energy shifts in the Si 2p or O 1s regions, while a gradually enhanced π‐π* shake‐up feature appears in the C 1s spectra, indicating the increasing contribution of graphitic sp^2^ domains introduced by 600JD and their role in promoting interfacial polarization.

### 2.2 Mechanical and Thermogravimetric Characteristics of 600JD‐Filled Silicone Rubber Composites

**Figure 3:**
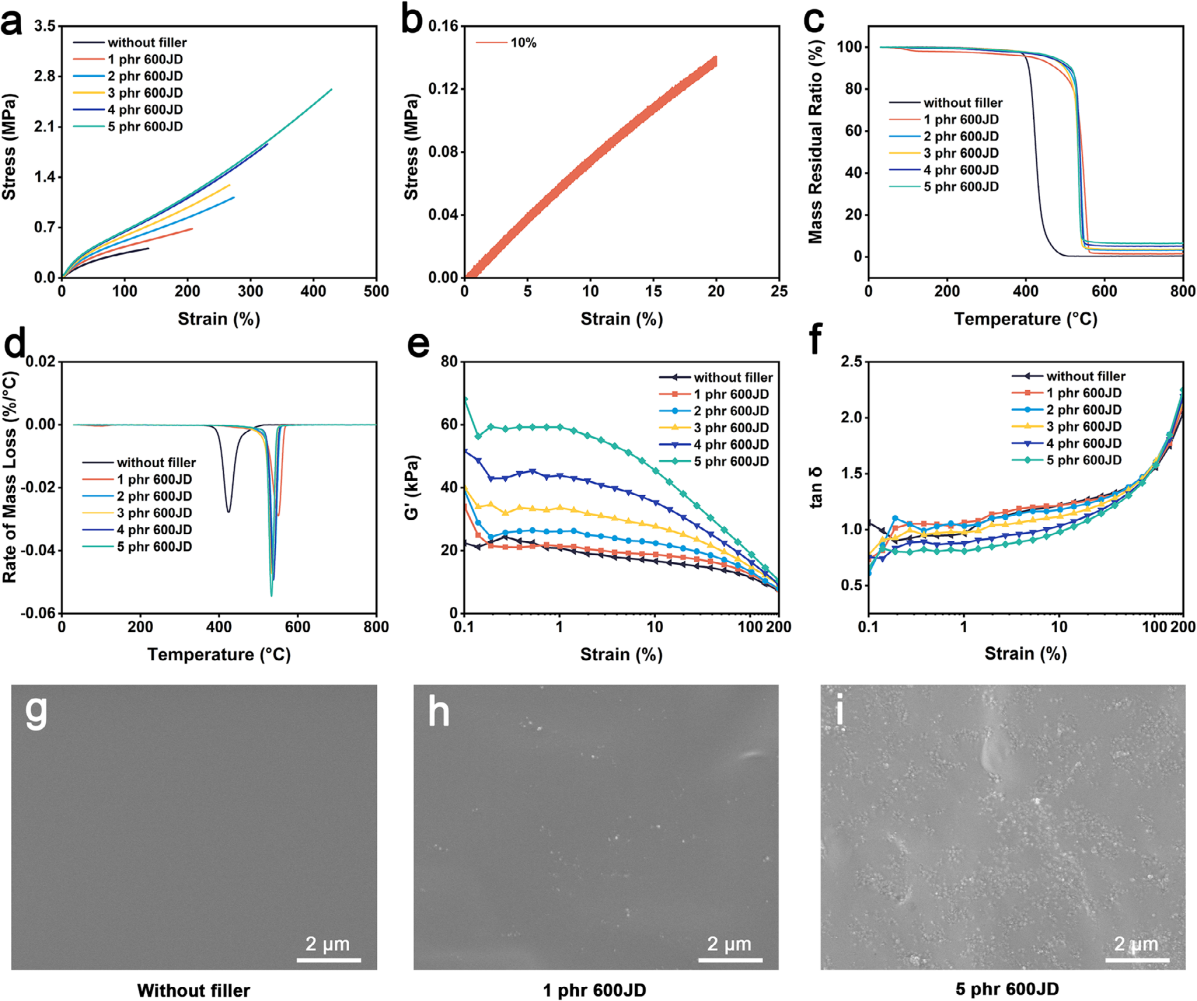
Mechanical and thermogravimetric characteristics of 600JD‐filled silicone rubber composites. a) Stress‐strain curves of composites with varying 600JD contents. b) Cyclic tensile response of the 1 phr 600JD‐filled sample under 10% strain. c) Mass residual ratio and d) Rate of mass loss from TGA tests of the composites. e) Storage modulus (*G'*) results and f) tan δ results from RPA tests of the composites. Cross‐sectional SEM image of g) the pristine silicone rubber, h) composite filled with 1 phr 600JD, and i) composite filled with 5 phr 600JD.

To evaluate the mechanical reinforcement effect of 600JD, tensile tests were performed on silicone rubber composites with varying filler loadings at a strain rate of 500 mm·min^−1^. As shown in **Figure** **3**a, the tensile strength and elastic modulus exhibited a monotonic increase with increasing 600JD content, with the 5 phr sample achieving the highest reinforcement level. To further assess elasticity and fatigue resistance, cyclic tensile tests were conducted on the 1 phr composite at multiple strain levels (10%, 20%, 50%, and 100%). The loading‐unloading curves demonstrated excellent elastic recovery and minimal hysteresis, even at large deformations, as shown in Figure S6 (Supporting Information). Moreover, under 40 repeated cycles (10% strain, 2 mm·min^−1^), the stress‐strain response remained stable without observable mechanical degradation (Figure 3b), confirming excellent fatigue durability under small‐strain cyclic loading.

Thermogravimetric analysis (TGA) was carried out to investigate the thermal stability of the composites under a nitrogen atmosphere at a heating rate of 10 °C·min^−1^. As shown in Figure 3c,d, the pristine silicone rubber displayed an initial decomposition temperature (*T_5%_*) of 399 °C. Upon incorporation of 600JD, both *T_5%_* and the residual char content progressively increased with filler content. Specifically, *T_5%_* increased from 420 °C (1 phr) to 473 °C (5 phr), while char yield rose from 1.5% to 6.6%. The enhanced thermal stability is attributed to the intrinsic high‐temperature resistance and barrier effect of carbon black particles, which suppresses heat and mass transfer during decomposition. Derivative thermogravimetry (DTG) curves showed that the peak degradation temperature (*T_max_*) shifted from 533 °C to 549 °C with increasing filler content, accompanied by a decrease in peak degradation rate. These results suggest that 600JD delays thermal decomposition and slows degradation kinetics, yielding a more thermally stable composite material.

To assess the filler dispersion and its effect on dynamic viscoelasticity, strain‐sweep tests were performed using a rubber process analyzer (RPA). As illustrated in Figure 3e, all composites exhibited a typical Payne effect, characterized by a gradual decrease in storage modulus (*G′*) with increasing strain, indicating the breakdown of the filler‐filler network under dynamic loading. As can be seen in Figure 3f, the corresponding tan δ‐strain curves showed low damping behavior at small strains, suggesting strong filler‐matrix interactions and a dominantly elastic response. At larger strains, tan δ increased, indicating enhanced energy dissipation due to polymer chain mobility and partial network disruption. These results confirm uniform dispersion of 600JD in the silicone matrix and its positive contribution to the nonlinear viscoelastic behavior.

Scanning electron microscopy (SEM) images of the pristine silicone rubber (Figure 3g), the composite with 1 phr of 600JD (Figure 3h), and the one with 5phr of 600JD (Figure 3i) reveal that 600JD is uniformly dispersed within the silicone rubber matrix at the nanoscale level, which aligns well with the observations from the RPA analysis. This group of samples was also characterized using transmission electron microscopy (TEM). As shown in Figure S7 (Supporting Information), 600JD exhibits a relatively uniform distribution within the silicone matrix. To identify the optimal counter‐material for triboelectric interaction, the composite containing 1 phr 600JD was tested against 14 different materials. As shown in Figure S8 (Supporting Information), polyvinylidene fluoride (PVDF) delivered the highest surface charge output, highlighting it as the most effective triboelectric pair material for the composite.

### 2.3 Electrical Output of the Spring‐Assisted TENG

**Figure 4:**
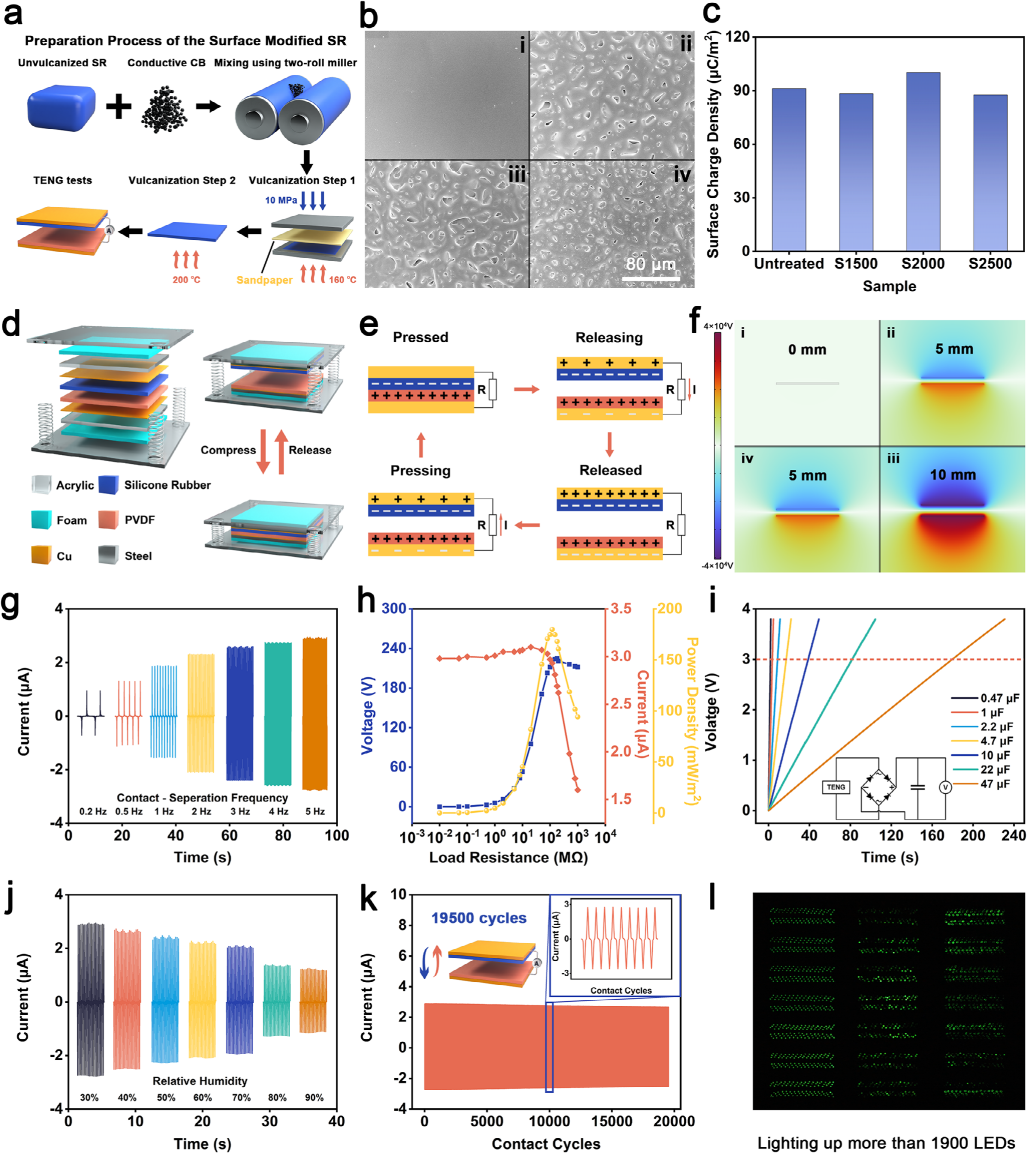
Electrical output of the spring‐assisted contact‐separation mode TENG constructed with surface‐treated silicone rubber. a) Schematic illustration of the preparation process for silicone rubber samples with surface microstructures. b) SEM images of untreated silicone rubber and samples treated with sandpapers of three different grit sizes: (i)untreated (ii)1500‐grit (iii)2000‐grit (iv)2500‐grit. c) Surface charge density generated by contact electrification between the sandpaper‐treated silicone rubber and PVDF film. d) Schematic diagram of the spring‐assisted contact‐separation mode TENG. e) Working principle schematic. f) COMSOL simulation of the potential distribution during the contact electrification process between 1 phr 600JD‐filled silicone rubber and PVDF. g) Short‐circuit current of the TENG under different contact‐separation frequencies. h) Output of the TENG at varying external loads. i) Voltage‐time curves for charging capacitors of various capacitance values using the TENG. j) Short‐circuit current of the TENG under different relative humidity conditions. k) Short‐circuit current during 19500 contact‐separation cycles. l) Photograph showing the TENG successfully lighting up over 1900 LEDs.

The construction of surface microstructures via templating methods is commonly employed as an effective strategy to enhance the triboelectric performance of materials.^[^ ^52^ ^]^ Herein, to further enhance the triboelectric performance of the composite, microstructures were introduced onto the surface of the silicone rubber via sandpaper templating. This surface modification improved interfacial contact during triboelectric operation, thereby enhancing charge transfer. As illustrated in **Figure** **4**a, the silicone rubber matrix was compounded with 1 phr of 600JD, hydroxyl silicone oil, and bis(2,5‐dimethyl‐2,5‐di‐tert‐butylperoxy)hexane using a two‐roll mill, followed by a two‐step vulcanization process. During the initial curing stage, sandpaper was applied to one side of the mold to imprint surface microstructures, and final crosslinking was completed through high‐temperature vulcanization.

Three different sandpapers, 1500, 2000, and 2500 grit, were used to create surface features of varying roughness. SEM images in Figure 4b confirmed the successful formation of microstructures with micron‐scale features on all treated samples, in contrast to the smooth morphology of the untreated reference. Figure 4c presents the surface charge density generated by the untreated and surface‐treated samples when paired with PVDF. The samples treated with sandpapers of grit sizes 1500, 2000, and 2500 were named S1500, S2000, and S2500, respectively. Notably, S2000 exhibited a significant enhancement in surface charge density. The surface charge density of this sample after contact electrification with PVDF reached 100 µC·m^−2^, representing an approximate 10% increase compared to the untreated sample (91.2 µC·m^−2^). The measurement results of the open‐circuit voltage and short‐circuit current also exhibit the same trend, as shown in Figures S9 and S10 (Supporting Information). The surface of S2000 exhibited a well‐defined microstructure, as confirmed by confocal laser scanning microscopy, see Figure S11 (Supporting Information), and showed a three‐dimensional surface roughness Sa of 4.802 µm.

Based on this optimization, a spring‐assisted contact‐separation mode TENG was fabricated using the S2000 composite and PVDF as the triboelectric layers. To clarify the interfacial contact characteristics, the surface morphology of the PVDF film was characterized by SEM and confocal laser scanning microscopy, as shown in Figure S12 (Supporting Information). The structure of the TENG is presented in Figure 4d. And its basic working mechanism is illustrated in Figure 4e, and the corresponding surface potential distribution, obtained via finite element simulation, is shown in Figure 4f and Video S1 (Supporting Information). The electrical output of the TENG was evaluated using a linear motor. As shown in Figure 4g, the short‐circuit current increased progressively with rising contact‐separation frequencies. Specifically, when the frequency increased from 0.2 to 5 Hz, the short‐circuit current rose from 0.9 to 2.9 µA. Similarly, the open‐circuit voltage increased from 154 to 221 V, as shown in Figure S13 (Supporting Information). Output under external load conditions was also assessed, and the voltage, current, and instantaneous output power density were plotted in Figure 4h. The instantaneous output power curves under different load conditions can be found in Figure S14 (Supporting Information). The results indicated an optimal load impedance of ≈120 MΩ, with a peak instantaneous power density of 179.9 mW·m^−2^.

The TENG's ability to charge capacitors of various capacitances was tested, and the corresponding voltage profiles are shown in Figure 4i. As expected, the charging time increased with larger capacitances. For example, charging a 10 µF capacitor required 38 s, while a 47 µF capacitor took ≈180 s to reach the same voltage. Overall, the TENG demonstrated good performance in capacitor charging. The effect of ambient humidity on the TENG's output was also investigated. As shown in Figure 4j, the short‐circuit current decreased from ≈2.9 to 1.2 µA as the relative humidity increased from 30% to 90%. The open‐circuit voltage showed a similar decreasing trend with increasing humidity (from 221 to 95 V), as illustrated in Figure S15 (Supporting Information). Long‐term stability tests of the TENG were conducted, and the results are plotted in Figure 4k. Even after 19500 cycles of contact and separation, the TENG maintained stable electrical output, confirming its excellent operational durability. Finally, we demonstrated the practical application of the TENG by successfully lighting over 1900 commercial LEDs, as shown in Figure 4l and Video S2 (Supporting Information), confirming its strong output performance and real‐world potential.

### 2.4 Gas Barrier and Flame‐Retardant Properties of the Encapsulation Material

As demonstrated in Figure 4j, the electrical output of the spring‐assisted contact‐separation mode TENG was markedly influenced by ambient humidity. To mitigate the detrimental effects of moisture and environmental interference, a multifunctional encapsulation layer with enhanced gas barrier and flame‐retardant properties was developed. CIIR, well known for its low gas permeability, was chosen as the matrix. To impart flame retardancy and improve barrier properties, a phosphorus‐nitrogen intumescent flame‐retardant system comprising aluminum diethylphosphinate (ADP), melamine cyanurate (MCA), and resorcinol bis(diphenyl phosphate) (RDP) was incorporated. Organically modified nanoclay was further introduced to enhance char‐forming ability and thermal stability. Detailed formulations are provided in Table S1 (Supporting Information). Seven CIIR‐based composite formulations, labeled CIIR‐0 through CIIR‐6, were prepared. All samples contained 20 phr of N330 carbon black as a reinforcing filler. CIIR‐0 was the unfilled control. CIIR‐1 to CIIR‐3 incorporated a phosphorus‐nitrogen flame retardant system composed of ADP, MCA, and RDP. Their mass ratios were 2:1:0.5 (CIIR‐1), 4:2:1 (CIIR‐2), and 6:3:1 (CIIR‐3), respectively, corresponding to total loadings of 35, 70, and 100 phr. CIIR‐4 to CIIR‐6 were formulated based on CIIR‐3, with additional organoclay introduced at 5, 10, and 15 phr, respectively.

**Figure 5:**
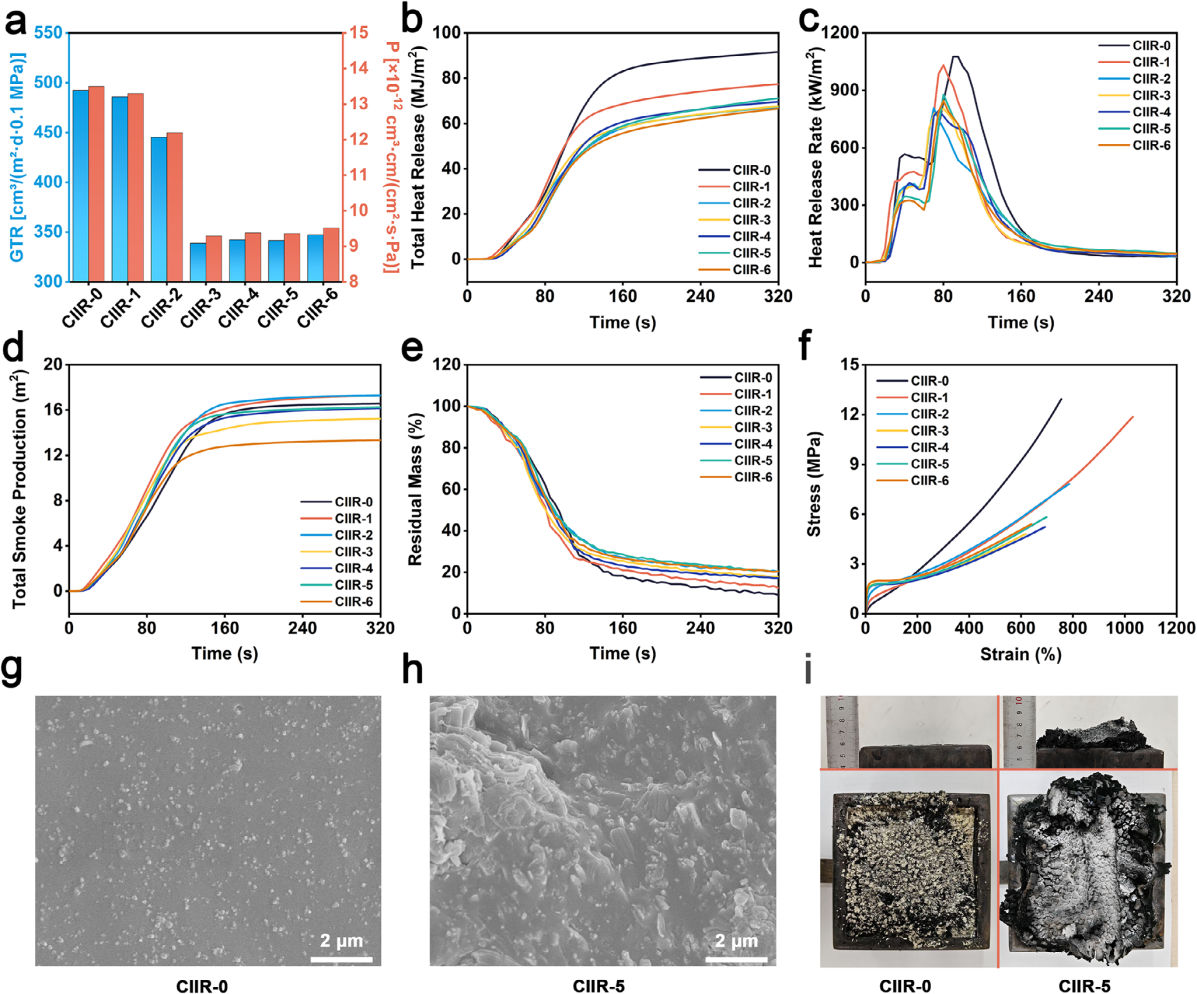
Gas barrier and flame‐retardant properties of the encapsulation material. a) P and GTR of the blank sample and the experimental group CIIR composites, b) THR, c) HRR, d) TSP, and e) Residual mass during the combustion test. f) Stress‐strain curve. Cross‐sectional SEM image of g) CIIR‐0 and h) CIIR‐5. i) Side view (top) and top view (bottom) of CIIR‐0 and CIIR‐5 samples after combustion.

Gas barrier properties were evaluated by measuring the gas transmission rate (GTR) and permeability coefficient (P), as can be seen in **Figure** **5**a. CIIR‐0 exhibited favorable gas barrier performance, with a GTR of 492.4 cm^3^/(m^2^·d·0.1 MPa) and a P of 1.35×10^−11^ cm^3^·cm/(cm^2^·s·Pa). As the loading of flame retardants increased from CIIR‐1 to CIIR‐3, both GTR and P decreased, indicating that the added fillers not only impart flame‐retardant functionality but also contribute to enhanced gas barrier properties. Notably, CIIR‐3 showed the best performance, with a GTR of 338.8 cm^3^/(m^2^·d·0.1 MPa) and a P of 9.29 × 10^−12^ cm^3^·cm/(cm^2^·s·Pa). This improvement is attributed to the physical blocking effect of ADP and MCA microcrystals, which occupy free volume and hinder gas diffusion pathways. Although these fillers lack the long‐range order of nanosheets, their high content results in effective tortuosity enhancement. Further addition of organoclay (CIIR‐4 to CIIR‐6) did not significantly enhance gas barrier properties beyond CIIR‐3, suggesting a saturation of barrier effects at high filler content. However, notable improvements in flame retardancy and smoke suppression were observed.

Cone calorimetry results in Figure 5b,c reveal reductions in both total heat release (THR) and peak heat release rate (pHRR) with increasing filler content. CIIR‐6 exhibited the lowest THR, confirming the synergistic effect of the phosphorus‐nitrogen system with organoclay. Smoke suppression performance, as shown in Figures 5d and S16 (Supporting Information), followed a similar trend. The total smoke production (TSP) and smoke production rate (SPR) values declined progressively from CIIR‐3 to CIIR‐6, with CIIR‐6 showing the best suppression. The improvement is linked to the high surface area and adsorptive nature of organoclay, which traps volatile degradation products and promotes stable char formation. Interestingly, CIIR‐1 and CIIR‐2 showed slightly higher TSP than the control, likely due to incomplete charring during initial decomposition, which led to the transient release of nitrogenous or aromatic volatiles. This effect was reversed at higher filler loadings, reinforcing the importance of sustained char formation. The gas and moisture barrier properties of the CIIR encapsulation layer benefit not only from the nanostructured organoclay, which increases the tortuous diffusion path of permeating molecules, but also from the intrinsic impermeability of the CIIR matrix itself. As a polar elastomer with low gas permeability, CIIR provides a reliable baseline for barrier performance. In addition, flame‐retardant additives such as ADP, MCA, and RDP are primarily introduced to enhance thermal safety by promoting the formation of protective char layers upon combustion, without compromising the sealing function of the encapsulation.

Figure 5e presents the char residue after combustion. CIIR‐0 retained only 9% mass, whereas CIIR‐5 and CIIR‐6 achieved 19%, reflecting improved thermal stability and carbon yield. Tensile behavior is shown in Figure 5f. While initial increases in filler loading reduced tensile strength and elongation at break due to matrix stiffening, further inclusion of organoclay (CIIR‐4 to CIIR‐6) partially restored flexibility. CIIR‐5 demonstrated a favorable balance, retaining 5.8 MPa strength and 700% elongation while achieving superior flame and barrier performance. Microstructural analysis via SEM, as shown in Figures 5g,h, and S17 (Supporting Information), revealed uniformly dispersed N330 particles in CIIR‐0 and a dense, tortuous layered network in CIIR‐5 due to intercalated clay platelets. This morphology effectively prolongs gas diffusion paths and reinforces char formation during combustion. Combustion residue morphology is presented in Figures 5i and S18 (Supporting Information). CIIR‐0 produced loose, powdery ash, whereas CIIR‐5 and CIIR‐6 formed continuous, cohesive char layers. The increased char height observed in cross‐sectional views indicates the swelling behavior of the intumescent system, further enhancing thermal insulation. The intrinsic flame retardancy of the CIIR encapsulation layer is essential for ensuring safety in practical deployment scenarios such as smart flooring. While its flame‐retardant nature primarily serves to comply with public safety requirements in polymer‐based infrastructures, CIIR's elastomeric characteristics further contribute to mechanical cushioning and interface stabilization. These features help enhance contact efficiency during the triboelectric process and reduce performance degradation caused by environmental aging, thereby indirectly supporting long‐term electrical stability. In summary, CIIR‐5 exhibited the most promising multifunctional performance, balancing gas impermeability, flame retardancy, smoke suppression, and mechanical reliability, making it a suitable candidate for TENG encapsulation in humid and harsh environments.

### 2.5 Performance of the Encapsulated TENG with Integrated Gas Barrier Layer

**Figure 6:**
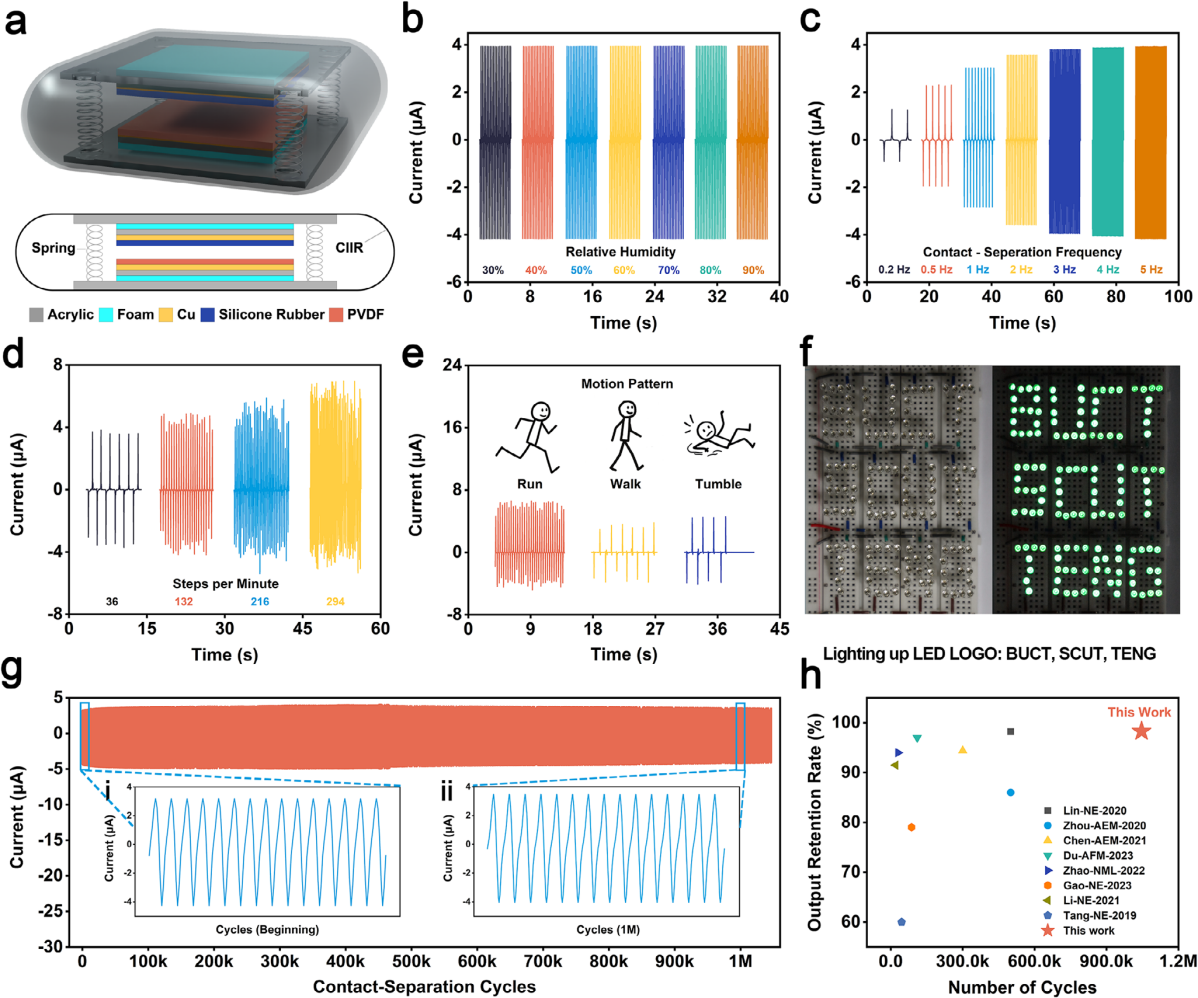
TENG performance with gas‐barrier encapsulation. a) Perspective and cross‐sectional views of the energy harvesting device. Short‐circuit current of the device b) under different humidity conditions, c) under different contact‐separation frequencies. d) under different stepping frequencies, and e) under different gait conditions. f) Demonstration of the device lighting up LEDs when stepped on. g) Short‐circuit current of the device after 1046000 contact‐separation cycles. h) Comparison of the output retention rate and cycling number of the device in this work with those reported in the literature.^[^ 56, 57, 58, 59, 60, 61, 62, 63 ^]^ The corresponding literature sources are indicated in the legend.

Based on the balance between gas barrier performance and mechanical properties, CIIR‐5 was identified as the optimal formulation and was employed as the encapsulation layer for the previously developed spring‐assisted contact‐separation TENG. This modification endowed the device with enhanced environmental stability. A schematic and cross‐sectional representation of the encapsulated device is provided in **Figure** **6**a. The encapsulated TENG's humidity resistance was assessed using a programmable linear motor. As shown in Figure 6b, the short‐circuit current remained nearly constant as the relative humidity increased from 30% to 90%, confirming the efficacy of the CIIR‐5 barrier layer in suppressing humidity‐induced degradation. Open‐circuit voltage plotted in Figure S19 (Supporting Information) showed similar stability.

The effect of contact‐separation frequency on output is presented in Figure 6c. As the frequency increased from 0.2 to 5 Hz, the short‐circuit current rose from 1.28 to 4.18 µA. The open‐circuit voltage exhibited a comparable increasing trend, increasing from 328 to 378 V as illustrated in Figure S20 (Supporting Information). In addition to controlled simulations, the device's performance was assessed under human motion. As depicted in Figure 6d, distinct short‐circuit current signals were generated during foot stepping. From the current waveforms, stepping frequencies corresponding to four different signal patterns were calculated as 36, 132, 216, and 294 steps per minute, respectively. Notably, higher stepping frequencies yielded higher current amplitudes, consistent with the frequency‐dependent behavior observed in linear motor simulations.

Notably, the encapsulated TENG exhibited motion pattern recognition capabilities. As illustrated in Figure 6e, different activities, including walking, running, and tumbling, produced characteristic current signatures, enabling the differentiation of user movement states. This highlights its potential as a self‐powered biomechanical sensor. To demonstrate practical functionality, commercial LED arrays forming the patterns “BUCT,” “SCUT,” and “TENG” were powered by the TENG during foot‐stepping, as shown in Figure 6f and Video S3 (Supporting Information). All patterns were simultaneously and brightly illuminated, underscoring the high instantaneous power output and real‐world utility of the device.

To evaluate the long‐term mechanical and electrical stability of the TENG device, a fatigue test exceeding 1 000 000 contact‐separation cycles was conducted. As shown in Figure 6g, the device maintained a consistent current output within about ±4 µA throughout the entire test period, with no apparent fluctuations or degradation. Representative current waveforms at the initial stage (Figure 6g‐i) and after 1 000 000 cycles (Figure 6g‐ii) show nearly identical peak amplitudes, frequencies, and waveform profiles. Quantitative analysis revealed a current retention rate of 98.3%, indicating negligible loss in charge transfer efficiency and excellent structural integrity of the triboelectric interface. These results confirm the outstanding durability and stability of the TENG under prolonged cyclic loading, underscoring its potential for long‐term use in practical applications such as footstep energy harvesting and intelligent sensing under variable environmental conditions.

To further elucidate the performance advantages of our device, a comparative analysis of recently reported HTV silicone rubber‐based TENG^[^ ^53^, ^54^, ^55^ ^]^ was conducted. As summarized in Table S2 (Supporting Information), our TENG, fabricated using only 1phr (0.97 wt.%) 600JD as filler, exhibits a notably high open‐circuit voltage (378 V) and short‐circuit current (7 µA), while achieving excellent charge storage capability (100 µC m^−2^). More importantly, the device demonstrates outstanding cyclic durability, maintaining stable output over 1 000 000 operating cycles, surpassing other HTV silicone rubber‐based systems, which typically fail to exceed 30 000 cycles. In contrast to previous studies that employed high filler loadings (up to 25–30 wt.%) or lacked moisture and flame resistance, our design offers an optimal balance of electrical output, environmental stability, and mechanical resilience. This comprehensive improvement highlights the practical viability and material‐level advancement achieved in our work.

To place this performance within the broader context of long‐lifespan TENGs, a comparative durability analysis was further conducted, as illustrated in Figure 6h. The red star marks this work, demonstrating a 98.3% output retention after 1 000 000 cycles. In contrast, prior studies such as Lin‐NE‐2020^[^ ^56^ ^]^ and Du‐AFM‐2023^[^ ^57^ ^]^ achieved slightly lower retention (>95%) at shorter cycle durations (<600 000), while others like Gao‐NE‐2023^[^ ^58^ ^]^ and Tang‐NE‐2019^[^ ^59^ ^]^ exhibited significant output degradation (<80%) at <200 000 cycles. The CIIR‐based encapsulation of the TENG not only provides gas and moisture barrier functionality but also offers intrinsic mechanical resilience and environmental resistance. Its elastic nature mitigates interfacial stress during repeated deformation, while the closed‐cell structure helps prevent dust and water vapor infiltration, thus enhancing long‐term operational reliability under practical conditions. These combined advantages ensure reliable long‐term operation under practical conditions, particularly for footstep energy harvesting systems subject to sustained mechanical deformation and environmental exposure.

## 3 Conclusion

In this study, we developed a resilient and scalable strategy for enhancing the triboelectric performance, environmental tolerance, and long‐term durability of silicone rubber‐based TENGs. By systematically incorporating six types of fillers into HTV silicone rubber, we identified 600JD as the most effective filler, achieving a 55% increase in surface charge density at only 1 phr loading. Based on this optimized formulation, a spring‐assisted contact‐separation mode TENG was constructed using 600JD‐filled silicone rubber and a PVDF film, achieving a peak power density of 179.9 mW·m^−2^ and successfully lighting over 1900 commercial LEDs.

To mitigate humidity‐induced performance degradation, a multifunctional encapsulation layer was engineered using CIIR with an integrated phosphorus‐nitrogen intumescent flame‐retardant system and organoclay reinforcement. The encapsulated TENG maintained stable output across a wide humidity range (30%–90% RH) and retained 98.3% of its initial current after over 1 000 000 cycles, confirming its excellent durability and environmental robustness. The CIIR‐based encapsulation system not only exhibits excellent environmental adaptability and intrinsic flame‐retardant properties but also benefits from mature industrial applications, high processing compatibility, and cost‐efficiency. Collectively, these advantages not only support scalable manufacturing but also establish a solid foundation for the practical deployment of TENGs in real‐world environments that demand both durability and multifunctional performance.

Beyond energy harvesting, the TENG exhibited real‐time biomechanical sensing capabilities, enabling accurate step frequency extraction and motion pattern recognition. These features underscore its potential in self‐powered systems for health monitoring and kinetic energy harvesting. Collectively, this work offers a scalable pathway for designing high‐performance, long‐lifetime TENGs capable of reliable operation in complex, real‐world environments, paving the way for their integration into next‐generation sustainable electronics. The integrated design demonstrates that industrially viable materials can be optimized for reliable, long‐term energy harvesting in real‐world environments.

## 4 Experimental Section

### Materials

Methyl vinyl silicone rubber (VMQ), bis(2,5‐dimethyl‐2,5‐di‐tert‐butylperoxy)hexane, hydroxyl‐terminated silicone oil, dielectric fillers (TiO_2_, BaTiO_3_, SrTiO_3_), fumed silica, carbon black (N220 and N330), conductive carbon black (EC‐600JD), chlorinated isobutylene‐isoprene rubber (CIIR), aluminum diethylphosphinate (ADP), melamine cyanurate (MCA), and resorcinol bis(diphenyl phosphate) (RDP) were used as received. Detailed specifications and suppliers are provided in Table S3 (Supporting Information).

### Preparation of the Filler‐Modified Silicone Rubber Composites

VMQ was compounded with the selected fillers and additives, hydroxyl‐terminated silicone oil, and bis(2,5‐dimethyl‐2,5‐di‐tert‐butylperoxy)hexane, on a two‐roll mill (HX‐8103‐6, Dongguan Hongxiang). The homogeneous compounds were subsequently subjected to a two‐step vulcanization process to yield rubber sheets with a uniform thickness of 1 mm. For samples with surface microstructures, textured sandpaper was positioned within the mold cavity during the initial curing stage, imprinting micro‐patterns on the composite surface.

### Characterization of the Silicone Rubber Composites

Tensile properties were measured using a Shimadzu AGX‐V universal testing machine. Surface potential measurements were conducted using Kelvin probe force microscopy (KPFM) on an atomic force microscope (Bruker, Dimension Icon). Surface morphology and filler dispersion were observed via field‐emission scanning electron microscopy (FE‐SEM, Hitachi S‐4800) and transmission electron microscopy (TEM, Hitachi H‐9500). Thermogravimetric analysis (TGA) was performed under nitrogen atmosphere at a heating rate of 10 °C·min^−1^ using the STARe TGA system (Mettler Toledo). Dynamic viscoelastic behavior was evaluated using a Rubber Process Analyzer (Alpha Technologies).

### Preparation and Characterization of the Gas‐Barrier Layer

CIIR‐based gas‐barrier composites were prepared via conventional rubber mixing with various combinations of aluminum diethylphosphinate (ADP), melamine cyanurate (MCA), resorcinol bis(diphenyl phosphate) (RDP), and organoclay. Cone calorimetry tests (CCTs) were performed on 100 × 100 × 3 mm^3^ specimens in accordance with ISO 5660, using a cone calorimeter (Fire Testing Technology Ltd.) under a constant external heat flux of 50 kW·m^−2^.

### Assembly of the TENG

The TENG device was constructed in a spring‐assisted contact‐separation mode using a 6 × 6 cm^2^ 600JD‐filled silicone rubber film, PVDF film, Cu electrodes, elastic foam, compression springs, and acrylic substrates. For encapsulation, the assembled device was coated with flame‐retardant CIIR composites and bonded using an adhesive. During operation, the device was subjected to cyclic loading and unloading via four identical compression springs, generating a maximum vertical force of ≈80 N. This corresponds to a surface pressure of 22.2 kPa on the sample.

### Electrical Measurement of the TENG Output

Triboelectric output measurements were conducted using a Keithley 6517B electrometer, a ZX21G precision resistance box (Shanghai Dongmao), and a programmable linear motor (LinMot). Different frequencies were adopted for different testing purposes: for evaluating the triboelectric output of various conductive filler composites, a low frequency of 1 Hz was used. For frequency dependence studies, a range of 0.2–5 Hz (including 0.2, 0.5, 1, 2, 3, 4, and 5 Hz) was tested to assess the device's dynamic response. For standard electrical characterization (e.g., V_oc_, I_sc_), a consistent frequency of 5 Hz was employed. For long‐term durability testing (1 million cycles), a high frequency of 50 Hz was selected to accelerate fatigue evaluation while maintaining operational stability. The volunteer first signed the informed consent for further human activity experiments.

## Conflict of Interest

The authors declare no conflict of interest.

## Supporting information



Supporting Information

Supplemental Video 1

Supplemental Video 2

Supplemental Video 3

## Data Availability

The data that support the findings of this study are available in the supplementary material of this article.

## References

[1] Lee Y. H. , Science 2024, 383, ado4308. 10.1126/science.ado4308 38422146

[2] Q. Zhang , C. Xin , F. Shen , Y. Gong , Y. Zi , H. Guo , Z. Li , Y. Peng , Q. Zhang , Z. L. Wang , Energy Environ. Sci. 2022, 15, 3688.

[3] W. Wu , X. Cao , J. Zou , Y. Ma , X. Wu , C. Sun , M. Li , N. Wang , Z. Wang , L. Zhang , Adv. Funct. Mater. 2018, 29, 1806331.

[4] Hu J. , Iwamoto M. , Chen X. , Nano‐Micro Lett. 2023, 16, 7. 10.1007/s40820-023-01238-8 PMC10628068 37930592

[5] Chen B. , Wang Z. L. , Small 2022, 18, 2107034.

[6] X. Xu , Q. Wu , Y. Pang , Y. Cao , Y. Fang , G. Huang , C. Cao , Adv. Funct. Mater. 2021, 32, 2107896.

[7] X. Wen , W. Yang , Q. Jing , Z. L. Wang , ACS Nano 2014, 8, 7405. 24964297 10.1021/nn502618f

[8] X. Xiao , X. Zhang , S. Wang , H. Ouyang , P. Chen , L. Song , H. Yuan , Y. Ji , P. Wang , Z. Li , M. Xu , Z. L. Wang , Adv. Energy Mater. 2019, 9, 1902460.

[9] S. Wang , Y. Xie , S. Niu , L. Lin , Z. L. Wang , Adv. Mater. 2014, 26, 2818. 24449058 10.1002/adma.201305303

[10] J. Wang , L. Pan , H. Guo , B. Zhang , R. Zhang , Z. Wu , C. Wu , L. Yang , R. Liao , Z. L. Wang , Adv. Energy Mater. 2019, 9, 1802892.

[11] Zhang C. , Zhou L. , Cheng P. , D. Liu , C. Zhang , X. Li , S. Li , J. Wang , Z. L. Wang , Adv. Energy Mater. 2021, 11, 2003616.

[12] L. Xu , T. Jiang , P. Lin , J. J. Shao , C. He , W. Zhong , X. Y. Chen , Z. L. Wang , ACS Nano 2018, 12, 1849. 29328629 10.1021/acsnano.7b08674

[13] M. A. M. Hasan , W. Zhu , C. R. Bowen , Z. L. Wang , Y. Yang , Nat. Rev. Electr. Eng. 2024, 1, 453.

[14] Chen B. , Yang Y. , Wang Z. L. , Adv. Energy Mater. 2018, 8, 1702649.

[15] Fan F.‐R. , Tian Z.‐Q. , L. W. Z. F. generator , Nano Energy 2012, 1, 328.

[16] Y. Yang , L. Zheng , J. Wen , F. Xing , H. Liu , Y. Shang , Z. L. Wang , B. Chen , Adv. Funct. Mater. 2023, 33, 2304366.

[17] X. Li , Z. Zhao , Y. Hu , Y. Gao , L. He , W. Qiao , B. Zhang , Y. Xu , Z. L. Wang , J. Wang , Energy Environ. Sci. 2024, 17, 1244.

[18] W. Wu , T. Yang , Y. Zhang , F. Wang , Q. Nie , Y. Ma , X. Cao , Z. L. Wang , N. Wang , L. Zhang , ACS Nano 2019, 13, 8202. 31244038 10.1021/acsnano.9b03427

[19] Z. Li , S. Zhang , H. Guo , B. Wang , Y. Gong , S. Zhong , Y. Peng , J. Zheng , X. Xiao , Nano Energy 2023, 113, 108595.

[20] Wang Z. L. , Wang A. C. , Mater. Today 2019, 30, 34.

[21] Wang Z. L. , Mater. Today 2017, 20, 74.

[22] Q. Zheng , L. Xin , Q. Zhang , F. Shen , X. Lu , C. Cao , C. Xin , Y. Zhao , H. Liu , Y. Peng , J. Luo , H. Guo , Z. Li , Adv. Mater. 2025, 37, 2417380. 10.1002/adma.202417380 39775869

[23] W. Tang , Q. Sun , Z. L. Wang , Chem. Rev. 2023, 123, 12105. 37871288 10.1021/acs.chemrev.3c00305 PMC10636741

[24] Z. Liu , X. Chen , Z. L. Wang , Adv. Mater. 2025, 37, 2409440. 10.1002/adma.202409440 39108037

[25] P. Yang , Z. Liu , S. Qin , J. Hu , S. Yuan , Z. L. Wang , X. Chen , Sci. Adv. 2024, 10, adr9139. 10.1126/sciadv.adr9139 PMC11661438 39705345

[26] Y. Wei , W. Wu , Y. Wang , X. Chen , Z. L. Wang , D. Yang , Adv. Funct. Mater. 2023, 33, 2213727.

[27] X. Cao , Y. Xiong , J. Sun , X. Xie , Q. Sun , Z. L. Wang , Nano‐Micro Lett. 2022, 15, 14. 10.1007/s40820-022-00981-8 PMC9768108 36538115

[28] D. Yang , Y. Ni , X. Kong , S. Li , X. Chen , L. Zhang , Z. L. Wang , ACS Nano 2021, 15, 14653. 34523330 10.1021/acsnano.1c04384

[29] J. Chu , W. Wu , Y. Wei , Z. L. Wang , X. Chen , L. Zhang , Adv. Funct. Mater. 2024, 34, 2402520.

[30] W. Wu , S. Wen , Y. Wei , L. Ruan , F. Li , X. Cao , Z. L. Wang , L. Zhang , Nano Energy 2023, 105, 108001.

[31] J. M. Donelan , Q. Li , V. Naing , J. A. Hoffer , D. J. Weber , A. D. Kuo , Science 2008, 319, 807. 18258914 10.1126/science.1149860

[32] Starner T. , IBM Syst. J. 1996, 35, 618.

[33] W. Yang , J. Chen , G. Zhu , J. Yang , P. Bai , Y. Su , Q. Jing , X. Cao , Z. L. Wang , ACS Nano 2013, 7, 11317. 24180642 10.1021/nn405175z

[34] Z. E. Yang , Y. Yang , F. Liu , Z. Wang , Y. Li , J. Qiu , X. Xiao , Z. Li , Y. Lu , L. Ji , Z. L. Wang , J. Cheng , ACS Nano 2021, 15, 2611. 33533242 10.1021/acsnano.0c07498

[35] L. Liu , M. Wu , W. Zhao , J. Tao , X. Zhou , J. Xiong , Adv. Funct. Mater. 2023, 34, 2308353.

[36] R. Umapathi , S. V. N. Pammi , S. Han , J. Haribabu , M. Safarkhani , G. M. Rani , Y. S. Huh , Chem. Eng. J. 2025, 511, 161799.

[37] R. Umapathi , M. Rethinasabapathy , V. Kakani , H. Kim , Y. Park , H. K. Kim , G. M. Rani , H. Kim , Y. S. Huh , Nano Energy 2025, 136, 110689.

[38] G. M. Rani , Pammi S. V. N. , Kim H. , H. S. Ahn , Y. C. Hu , J. H. Jung , Y. S. Huh , Mater. Horiz. 2025, 10.1039/D5MH01578B. 41186964

[39] G. M. Rani , H. Kim , S. V. N. Pammi , R. Umapathi , Y. S. Huh , Adv. Funct. Mater. 2025, 19594, 10.1002/adfm.202519594.

[40] Y. Sun , Y. Zheng , R. Wang , T. Lei , J. Liu , J. Fan , W. Shou , Y. Liu , Nano Energy 2022, 100, 107506.

[41] S. A. Graham , B. Dudem , H. Patnam , A. R. Mule , J. S. Yu , ACS Energy Lett. 2020, 5, 2140.

[42] A. R. Mule , B. Dudem , S. A. Graham , J. S. Yu , Adv. Funct. Mater. 2019, 29, 1807779.

[43] K. Luo , T. Peng , Y. Zheng , Y. Ni , P. Liu , Q. Guan , Z. You , Adv. Mater. 2024, 36, 2312500. 10.1002/adma.202312500 38215006

[44] H. Zhang , Y. Han , Q. Guan , Z. You , M. Zhu , Adv. Mater. 2024, 36, 2403908. 10.1002/adma.202403908 38828745

[45] X. Gao , F. Xing , F. Guo , J. Wen , H. Li , Y. Yang , B. Chen , Z. L. Wang , Mater. Today 2023, 65, 26.

[46] Z. Liu , X. Liang , H. Liu , Z. Wang , T. Jiang , Y. Cheng , M. Wu , D. Xiang , Z. Li , Z. L. Wang , L. Li , ACS Nano 2020, 14, 15458. 32991146 10.1021/acsnano.0c06100

[47] S.‐H. Shin , Y. E. Bae , H. K. Moon , J. Kim , S.‐H. Choi , Y. Kim , H. J. Yoon , M. H. Lee , J. Nah , ACS Nano 2017, 11, 6131. 28558185 10.1021/acsnano.7b02156

[48] Y. Liu , J. Mo , Q. Fu , Y. Lu , N.i Zhang , S. Wang , S. Nie , Adv. Funct. Mater. 2020, 30, 2004714.

[49] W. Seung , M. K. Gupta , K. Y. Lee , K.‐S. Shin , J.u‐H. Lee , T. Y. Kim , S. Kim , J. Lin , J. H. Kim , S.‐W. Kim , ACS Nano 2015, 9, 3501. 25670211 10.1021/nn507221f

[50] W. Song , B. Gan , T. Jiang , Y. Zhang , A. Yu , H. Yuan , N. Chen , C. Sun , Z. L. Wang , ACS Nano 2016, 10, 8097. 27494273 10.1021/acsnano.6b04344

[51] W. Seung , H.‐J. Yoon , T. Y. Kim , H. Ryu , J. Kim , J.‐H. Lee , J. H. Lee , S. Kim , Y. K. Park , Y. J. Park , S.‐W. Kim , Adv. Energy Mater. 2017, 7, 1600988.

[52] C. Cao , Z. Li , F. Shen , Q. Zhang , Y. Gong , H. Guo , Y. Peng , Z. L. Wang , Energy Environ. Sci. 2024, 17, 885.

[53] X. Hou , J. Zhu , J. Qian , X. Niu , J. He , J. Mu , W. Geng , C. Xue , X. Chou , ACS Appl. Mater. Interfaces 2018, 10, 43661. 30474951 10.1021/acsami.8b16267

[54] Y. Shao , C. Luo , B.‐W. Deng , B. Yin , M.‐B. Yang , Nano Energy 2020, 67, 104290.

[55] X. Hou , S. Zhang , J. Yu , C. Yang , N. Zhang , J. He , X. Chou , Sci. China‐Technol. Sci. 2021, 64, 662.

[56] Z. Lin , B. Zhang , H. Zou , Z. Wu , H. Guo , Y. Zhang , J. Yang , Z. L. Wang , Nano Energy 2020, 68, 104378.

[57] S. Du , S. Fu , W. He , Q. Li , K. Li , H. Wu , J. Wang , C. Shan , Q. Mu , C. Hu , Adv. Funct. Mater. 2023, 33, 2306491.

[58] M. Gao , S.‐B. Kim , Y. Li , S. H. Ramaswamy , J. Choi , Nano Energy 2023, 105, 107997.

[59] Q. Tang , X. Pu , Q. Zeng , H. Yang , J. Li , Y. Wu , H. Guo , Z. Huang , C. Hu , Nano Energy 2019, 66, 104087.

[60] Zhou L. , Liu D. , Zhao Z. , S. Li , Y. Liu , L. Liu , Y. Gao , Z. L. Wang , J. Wang , Adv. Energy Mater. 2020, 10, 2002920.

[61] Chen P. , An J. , Shu S. , R. Cheng , J. Nie , T. Jiang , Z. L. Wang , Adv. Energy Mater. 2021, 11, 2003066.

[62] J. Zhao , D. Wang , F. Zhang , J. Pan , P. Claesson , R. Larsson , Y. Shi , Nano‐Micro Lett. 2022, 14, 160. 10.1007/s40820-022-00903-8 PMC9356124 35930162

[63] M. Li , W.‐Y. Cheng , Y.‐C. Li , H.‐M. Wu , Y.‐C. Wu , H.‐W. Lu , S.‐L. Cheng , L. Li , K.‐C. Chang , H.‐J. Liu , Y.‐F. Lin , L.‐Y. Lin , Y.‐C. Lai , Nano Energy 2021, 79, 105405.

