# Supplementary material for: Ambient Stable Triboelectric Nanogenerator Based on Conductive Filler Modified Silicone Rubber with Gas Barrier Encapsulation for Footstep Energy Conversion
Source: Adv Sci (Weinh). 2025 Dec 17;13(10):e19523. doi: 10.1002/advs.202519523 (PMC12915142; doi:10.1002/advs.202519523)
Supplement: Supplementary file 1 — Supporting Information [file ADVS-13-e19523-s002.docx]

Supporting Information

Ambient Stable Triboelectric Nanogenerator Based on Conductive Filler Modified Silicone Rubber with Gas Barrier Encapsulation for Footstep Energy Conversion

Yi Wei^1#^, Yushu Tian^1#^, Xiaokang Chen^2^, Junfeng Chu^1^, Junjie Wang^1^, Xiangyu Chen^3*^, Wenjie Wu^2*^, Liqun Zhang^4^

Yi Wei and Yushu Tian contributed equally to this work

Yi Wei, Yushu Tian, Junfeng Chu, Junjie Wang

^1^State Key Laboratory of Organic-Inorganic Composites, College of Materials Science and Engineering, Beijing University of Chemical Technology, Beijing 100029, P.R. China

Xiaokang Chen, Wenjie Wu

^2^Institute of Emergent Elastomers, School of Materials Science and Engineering, South China University of Technology, Guangzhou 510640, P. R. China

E-mail: wuwenjie@scut.edu.cn

Xiangyu Chen

^3^Beijing Key Laboratory of Micro-nano Energy and Sensor, Beijing Institute of Nanoenergy and Nanosystems, Chinese Academy of Sciences, Beijing 100083, P. R. China

E-mail: chenxiangyu@binn.cas.cn

Liqun Zhang

^4^State Key Laboratory of Fluorine and Nitrogen Chemicals, School of Chemical Engineering and Technology, Xi’an Jiao Tong University, Xi’an, Shaanxi 710049, P. R. China


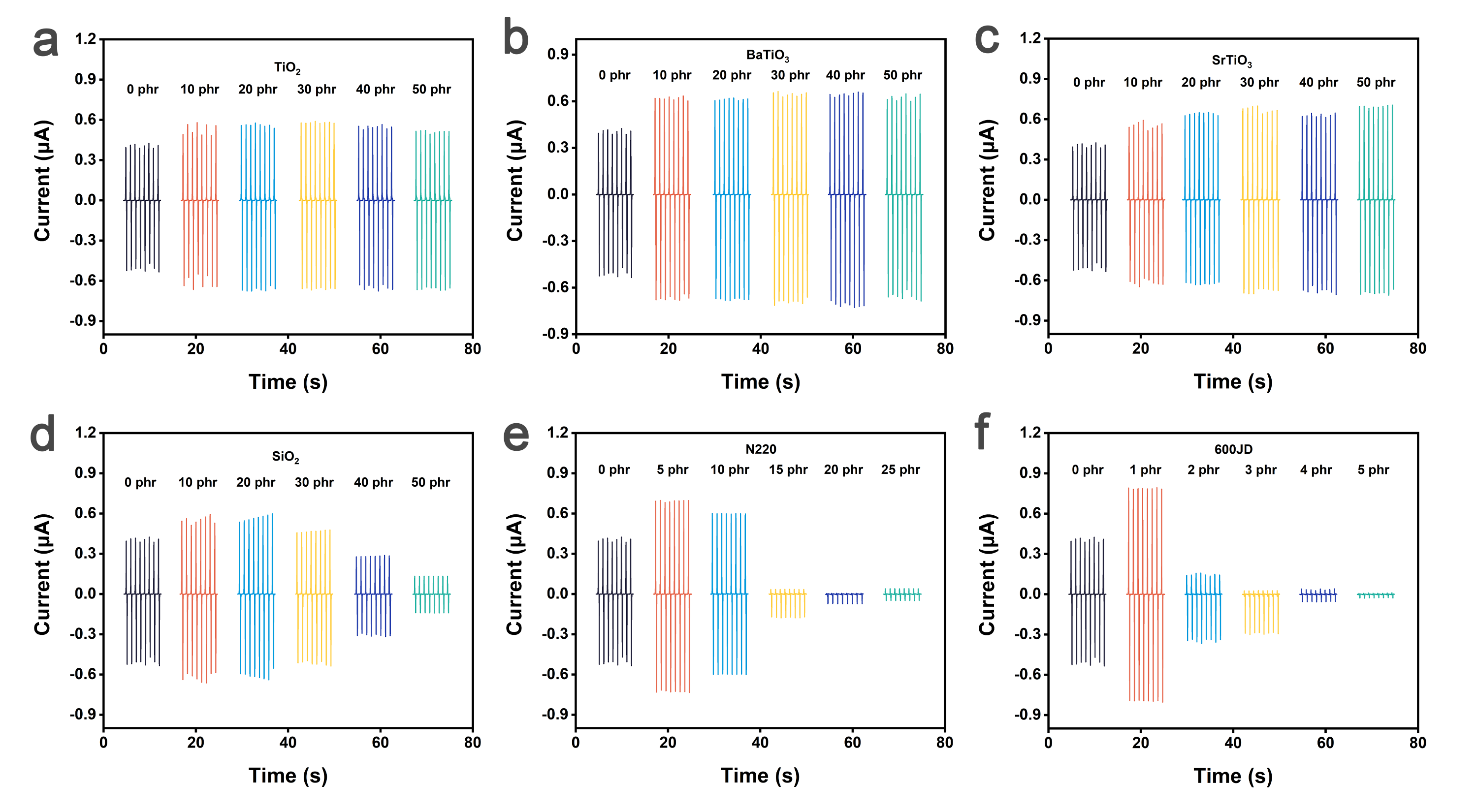


**Figure S1.** Short-circuit current of silicone rubber composites filled with six different fillers: (a) TiO_2_, (b) BaTiO_3_, (c) SrTiO_3_, (d) SiO_2_, (e) N220, (f) 600JD.

**
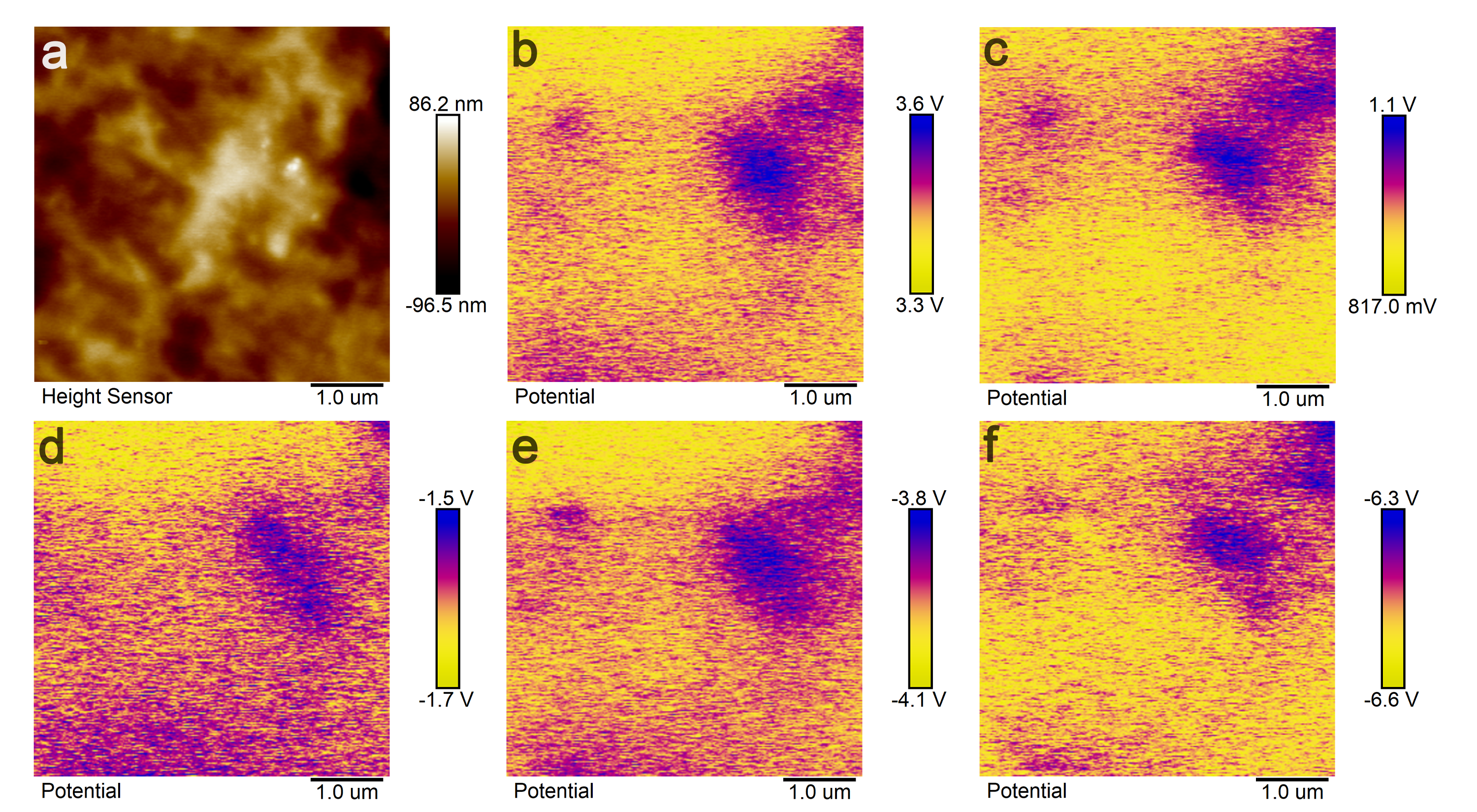
**

**Figure S2.** KPFM characterization results of the height and surface potential of 1 phr 600JD-filled silicone rubber under different bias voltages: (a) height, surface potential when bias is (b)5V, (c)2.5V, (d)0V, (e)-2.5V, (f)-5V.


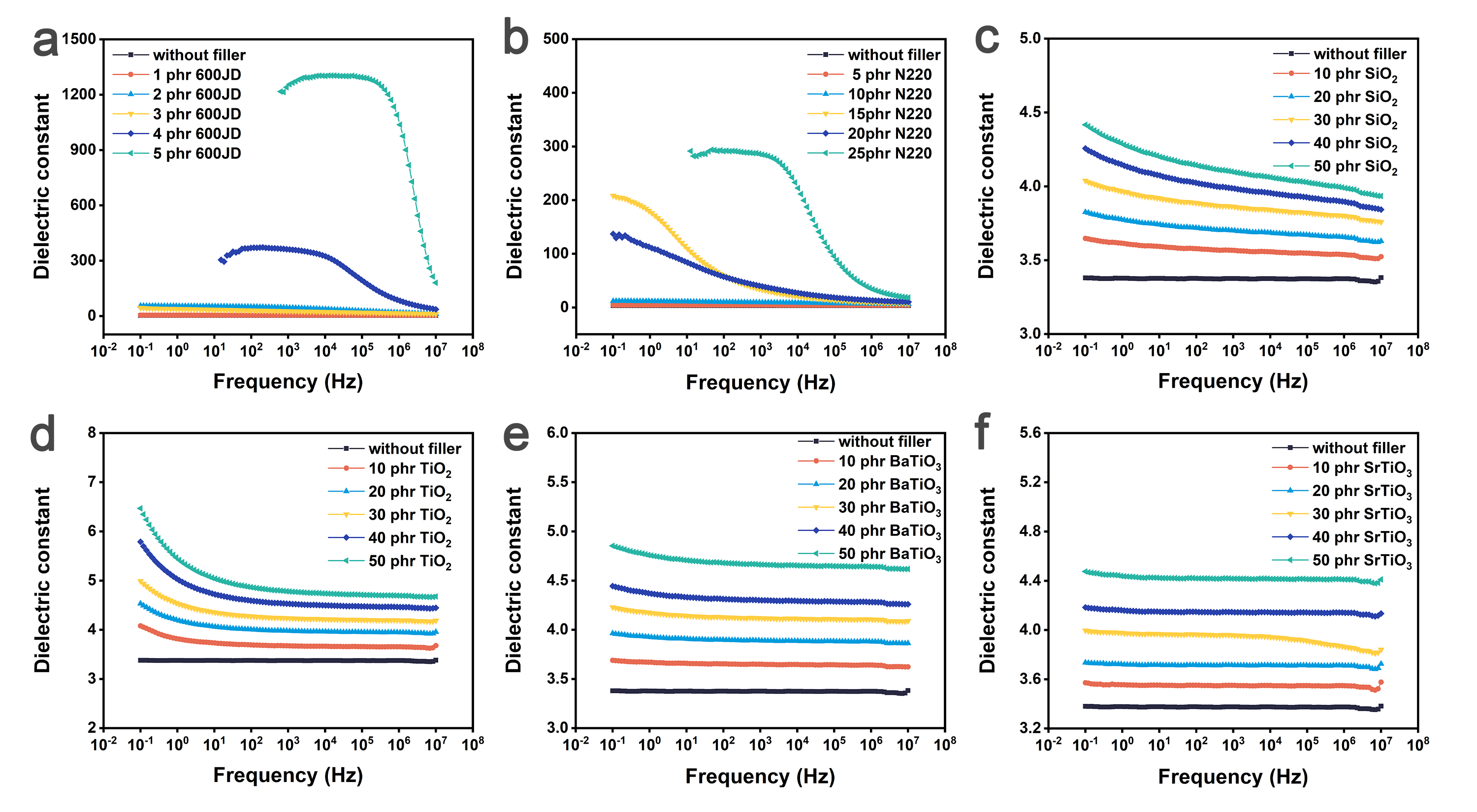


**Figure S3.** Frequency-dependent dielectric constant (ε′) of silicone rubber composites filled with different types and concentrations of fillers. (a) 600JD, (b) N220, (c) SiO_2_, (d) TiO_2_, (e) BaTiO_3_, and (f) SrTiO_3_. Note: For composites with higher filler loadings (600JD and N220), some data points in the low-frequency range (<1000 Hz) showed anomalous responses, possibly due to phase resolution limitations or instrument instability under high-loss conditions. These points were excluded to maintain clarity and consistency in data presentation.


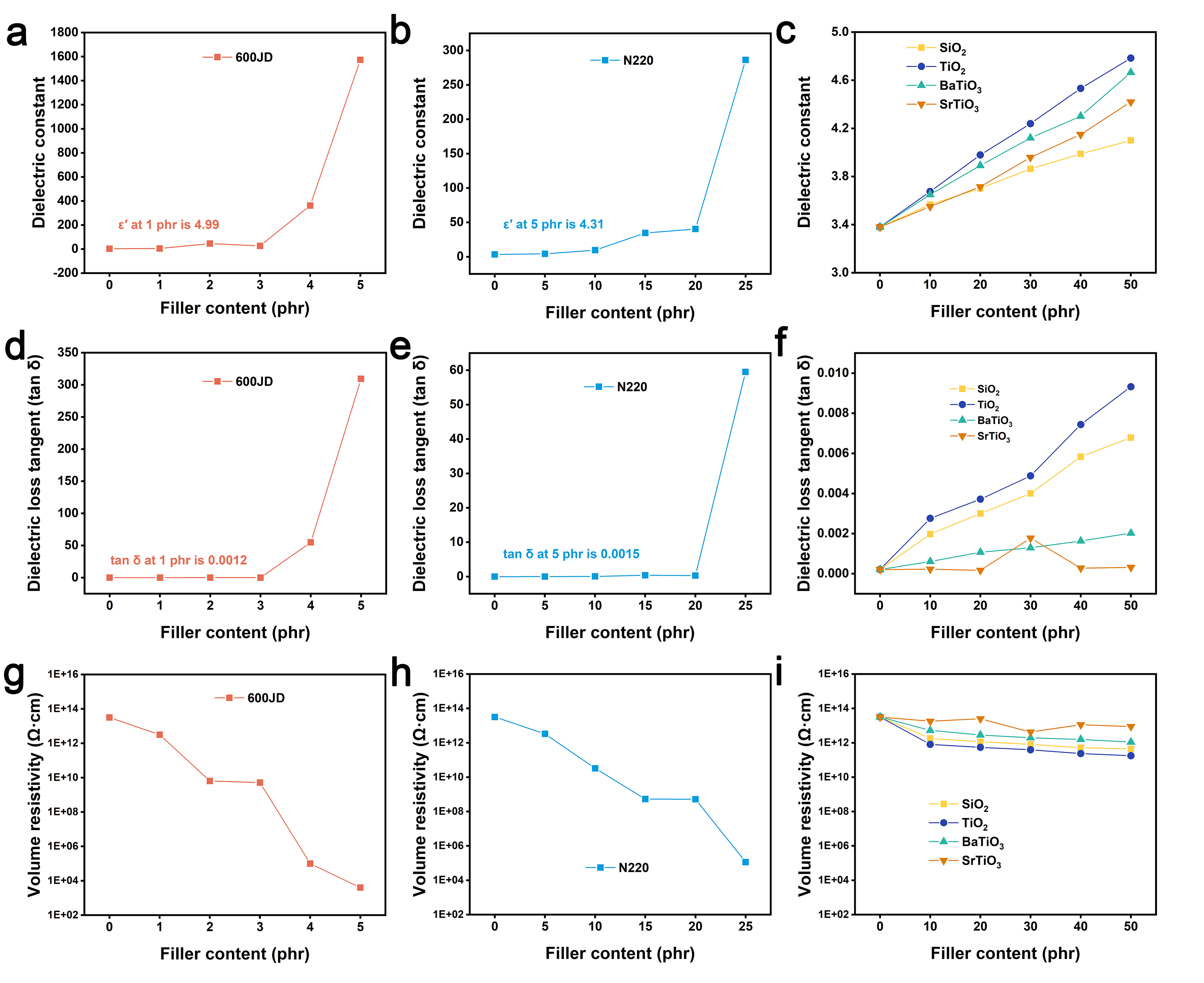


**Figure S4.** Dielectric and electrical properties of silicone rubber composites as a function of filler content for six types of fillers: Dielectric constant (ε′) measured at 1 kHz: (a) 600JD-filled composites, (b) N220-filled composites, (c) Composites filled with SiO_2_, TiO_2_, BaTiO_3_, and SrTiO_3_. Dielectric loss tangent (tan δ) measured at 1 kHz: (d) 600JD-filled composites, (e) N220-filled composites, (f) Composites filled with SiO_2_, TiO_2_, BaTiO_3_, and SrTiO_3_. Volume resistivity measured at 100 Hz: (g) 600JD-filled composites, (h) N220-filled composites, (i) Composites filled with SiO_2_, TiO_2_, BaTiO_3_, and SrTiO_3_.


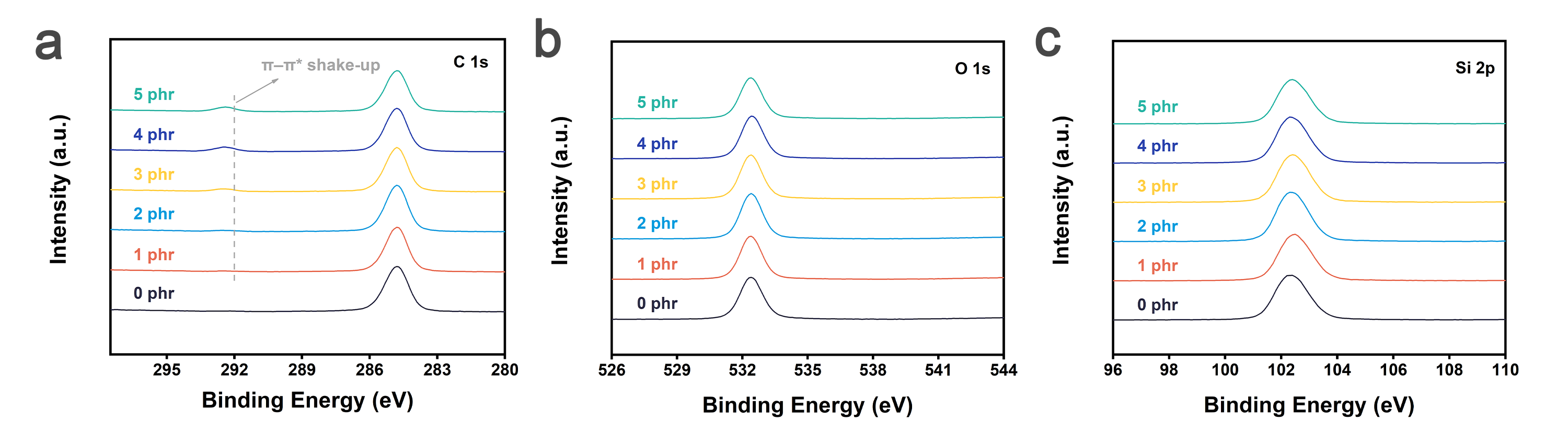


**Figure S5.** High-resolution XPS spectra of HTV silicone rubber composites with different loadings of 600JD carbon black. (a) C 1s spectra show a characteristic π-π* shake-up satellite (~292 eV), which becomes more visible with increasing filler content, indicating the introduction of graphitic/sp² carbon domains. (b) O 1s and (c) Si 2p spectra display no obvious chemical shift with filler addition, confirming that the silicone network remains chemically stable.


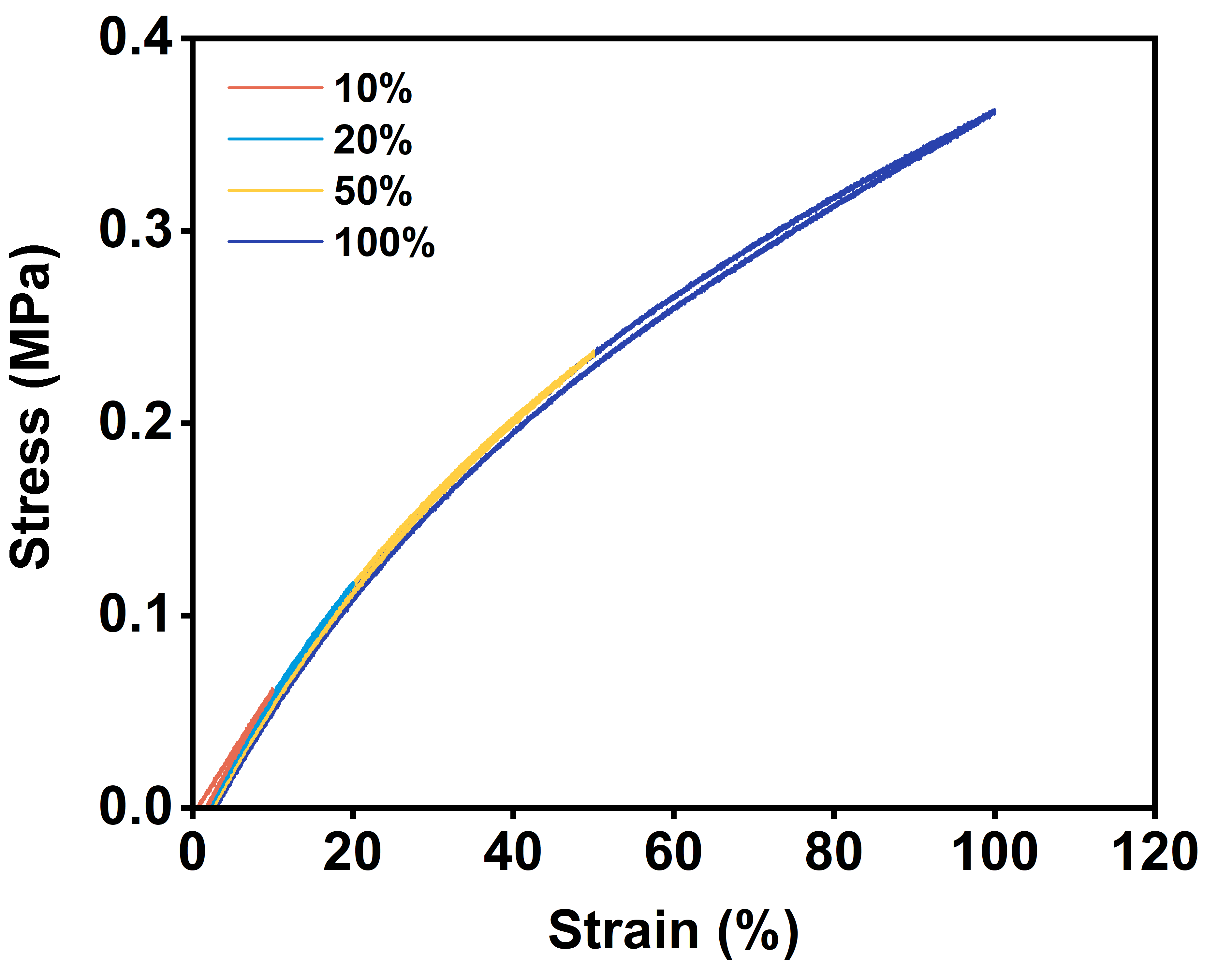


**Figure S6.** Cyclic tensile curves of silicone rubber filled with 1 phr 600JD under different strain levels.


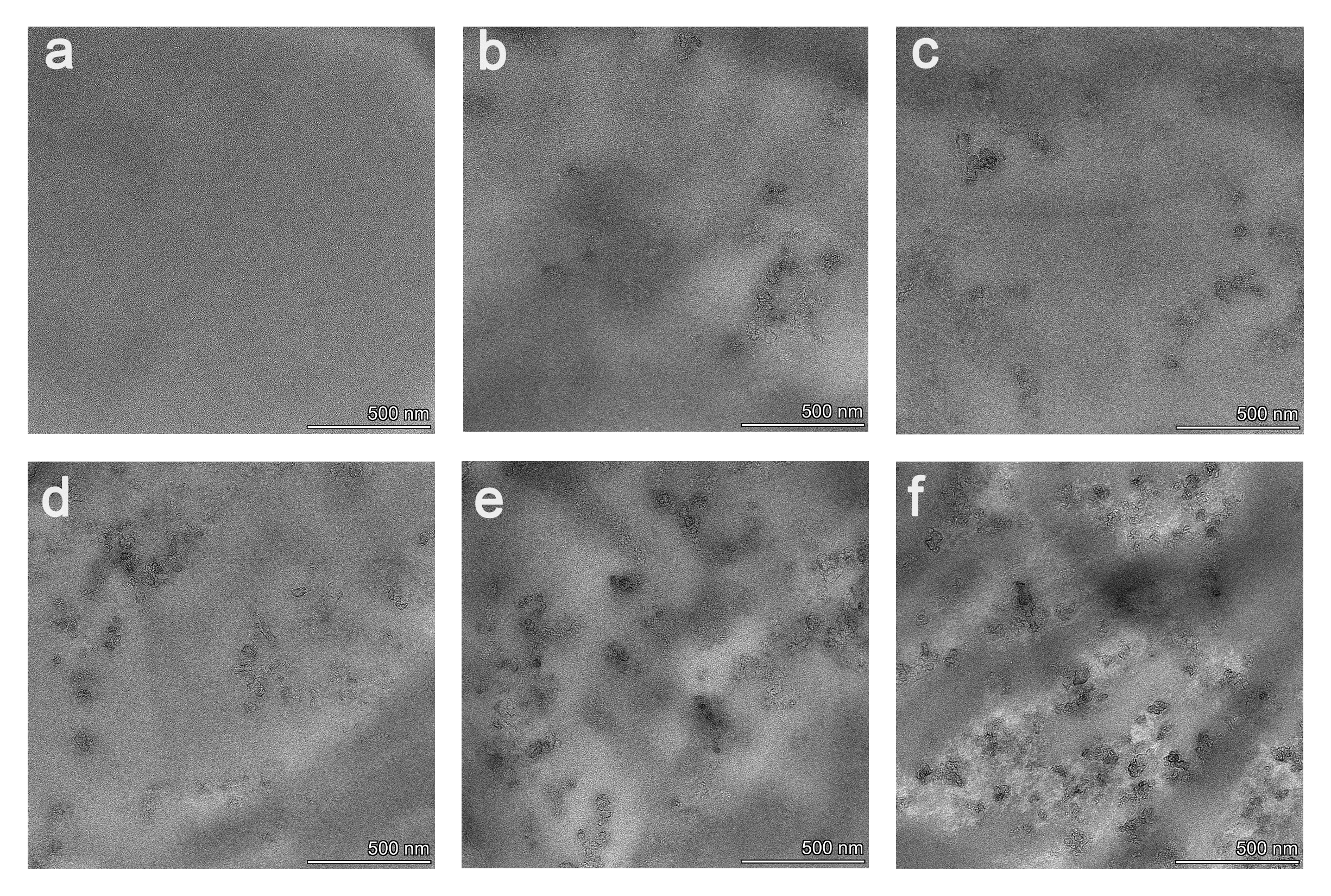


**Figure S7.** TEM images of the blank and 600JD-filled silicone rubber. (a) without 600JD, (b) 1 phr 600JD, (c) 2 phr 600JD, (d) 3 phr 600JD, (e) 4 phr 600JD, (f) 5 phr 600JD.


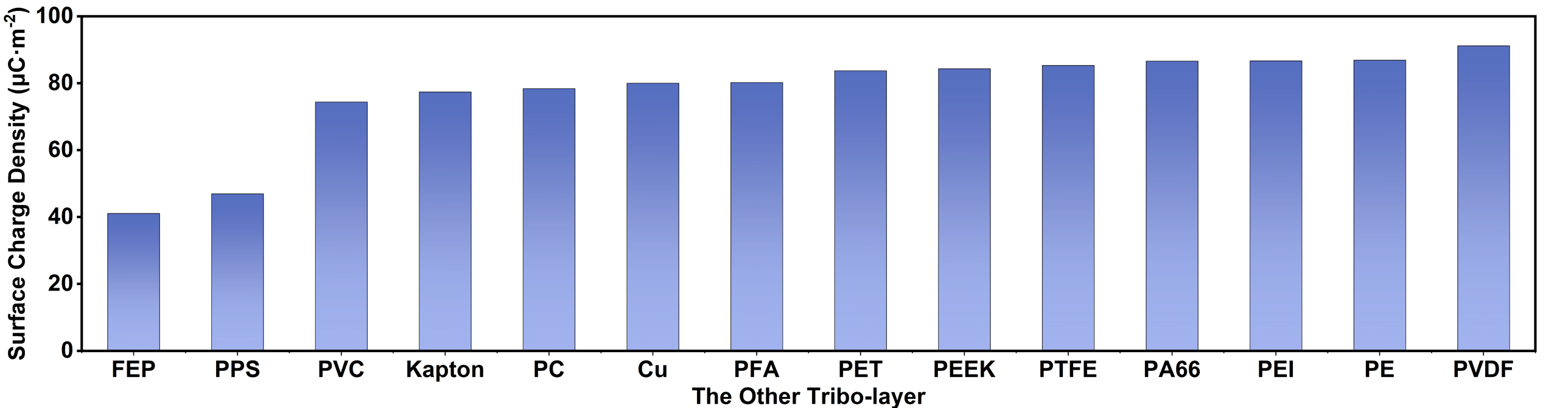


**Figure S8.** Surface charge density of silicone rubber filled with 1 phr 600JD after contact electrification with different materials


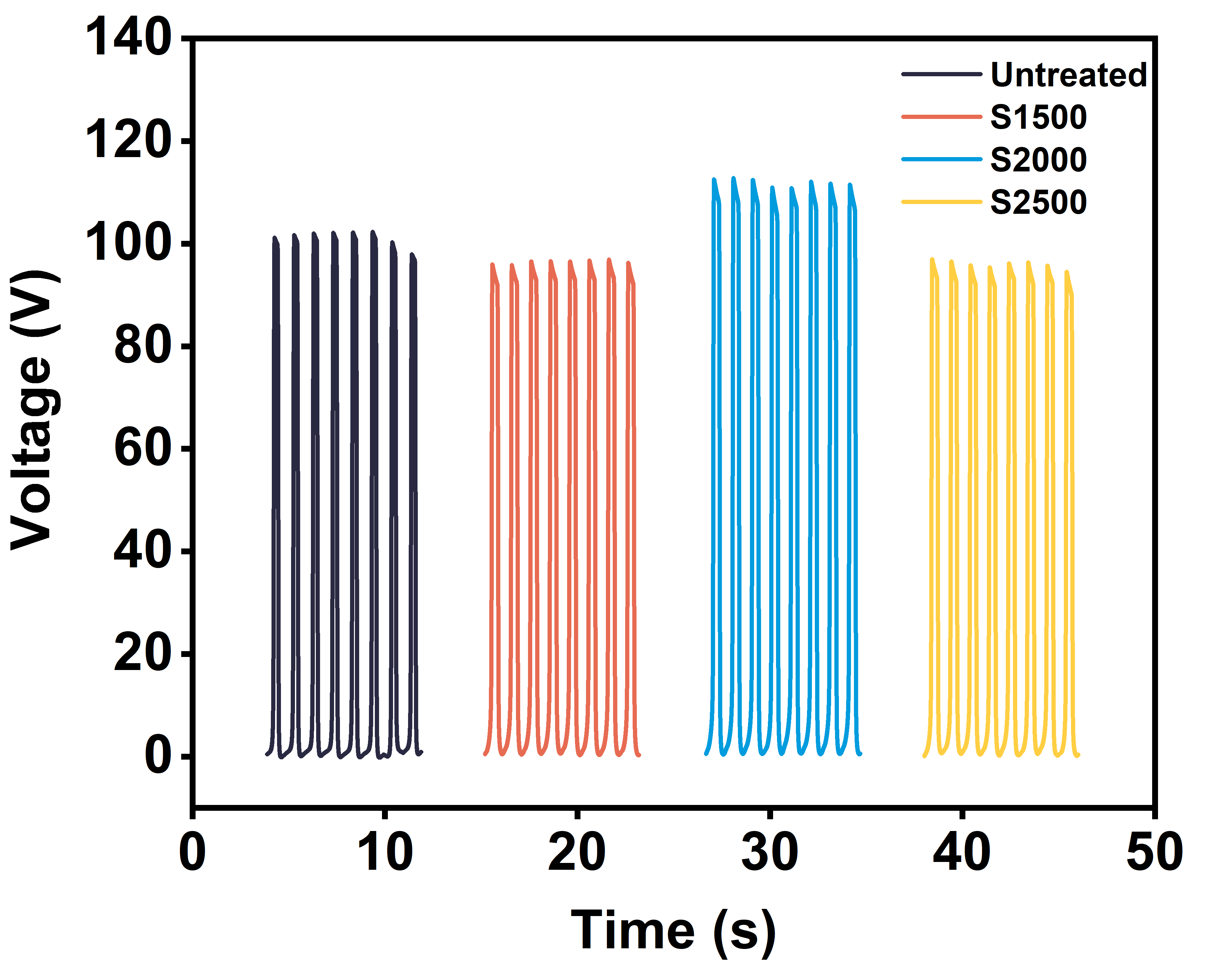


**Figure S9.** Open-circuit voltage generated by contact electrification between sandpaper-treated silicone rubber and PVDF film


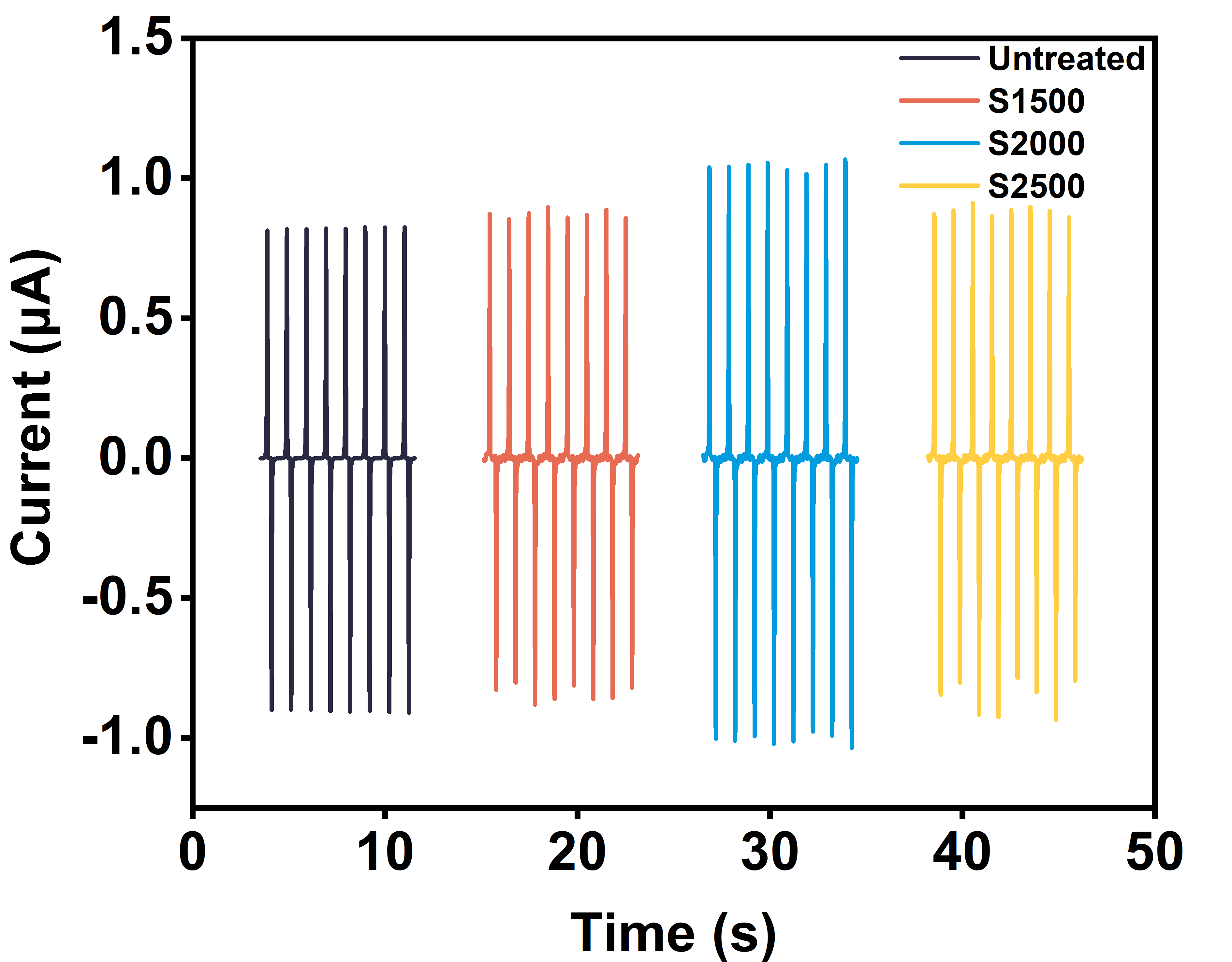


**Figure S10.** Short-circuit current generated by contact electrification between sandpaper-treated silicone rubber and PVDF film


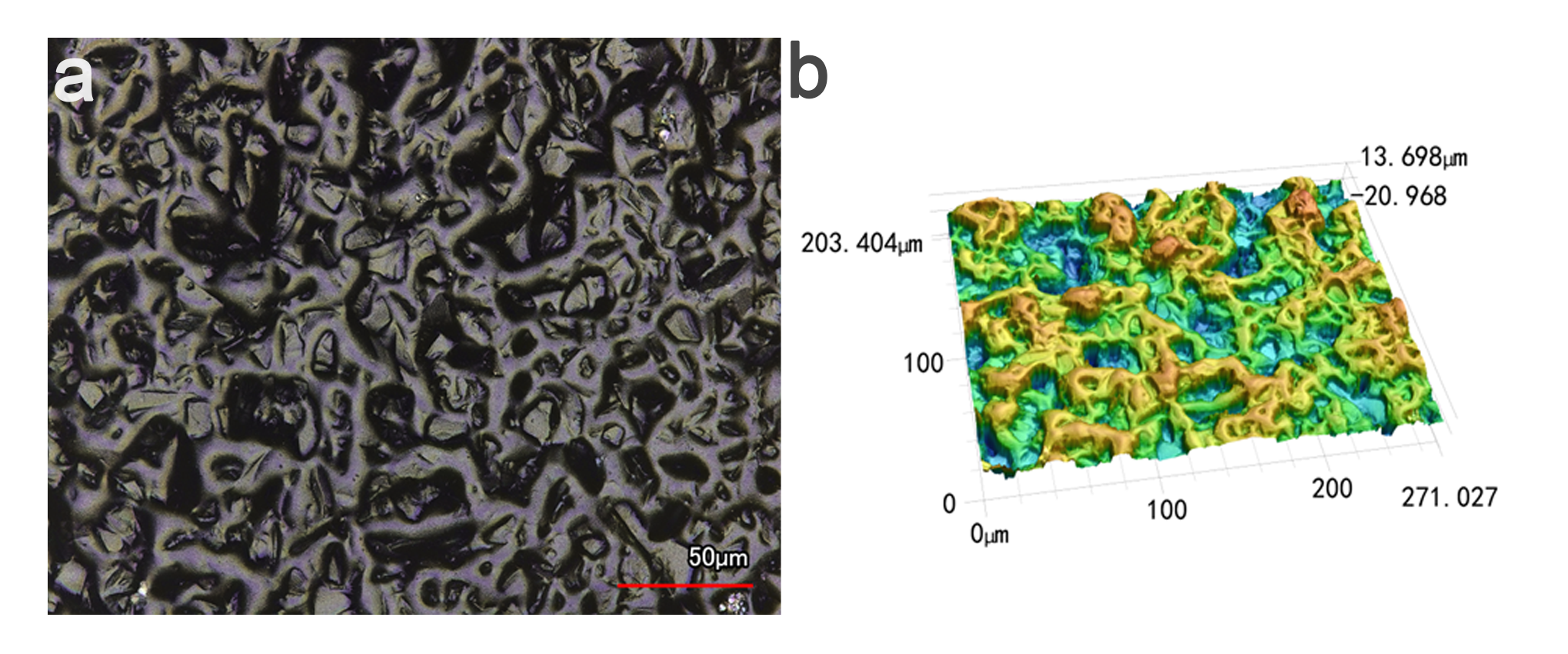


**Figure S11.** Confocal laser scanning microscopy images of the silicone rubber surface treated with 2000-grit sandpaper: (a) planar view and (b) 3D height map. The average surface roughness (Sa) is 4.802 µm.


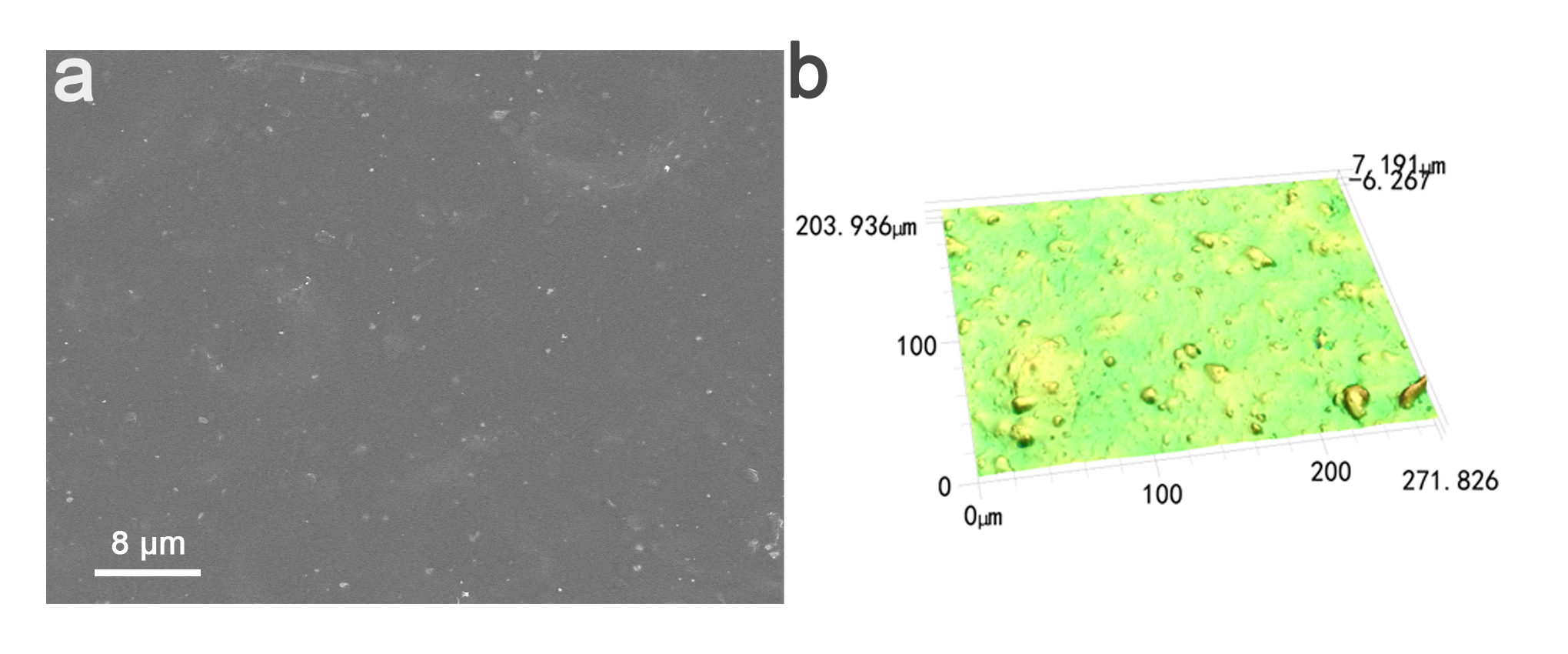


**Figure S12.** Surface morphology of the PVDF film. (a) SEM image of the commercial PVDF surface. (b) 3D CLSM topography of the PVDF film, with an average surface roughness (Sa) of 0.487 μm.


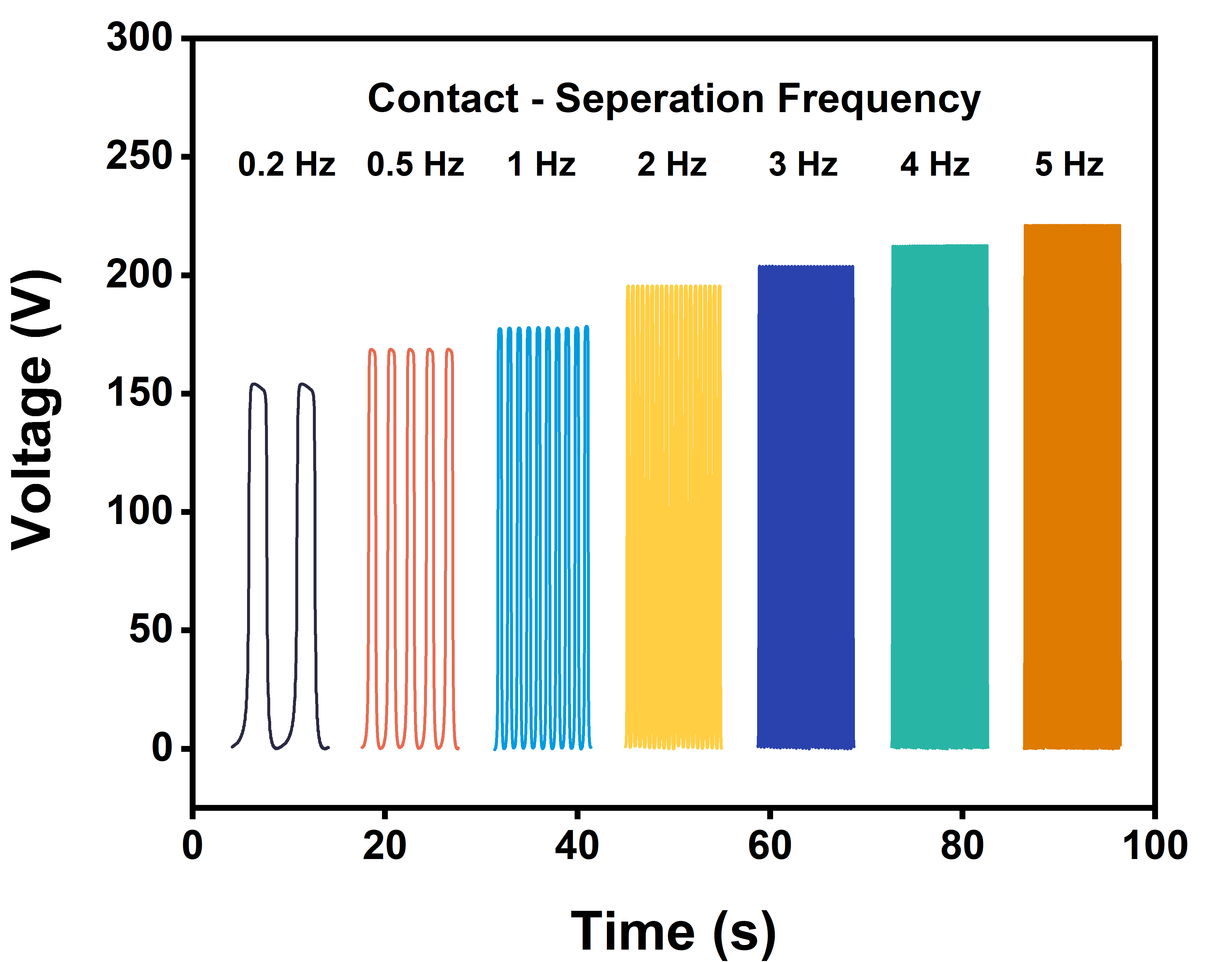


**Figure S13.** Open-circuit voltage of the TENG at different contact-separation frequencies


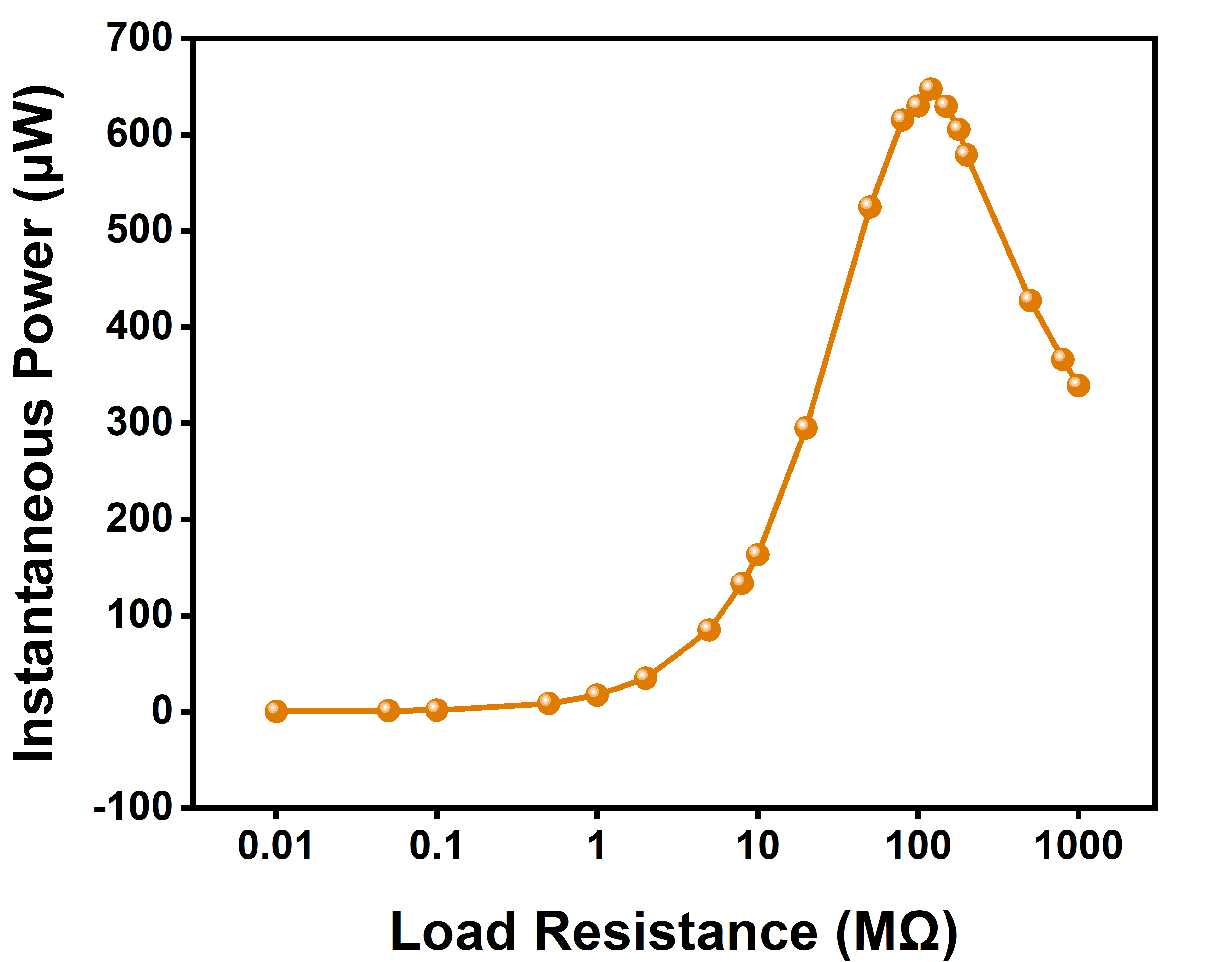


**Figure S14.** Instantaneous power of the TENG under varying external loads


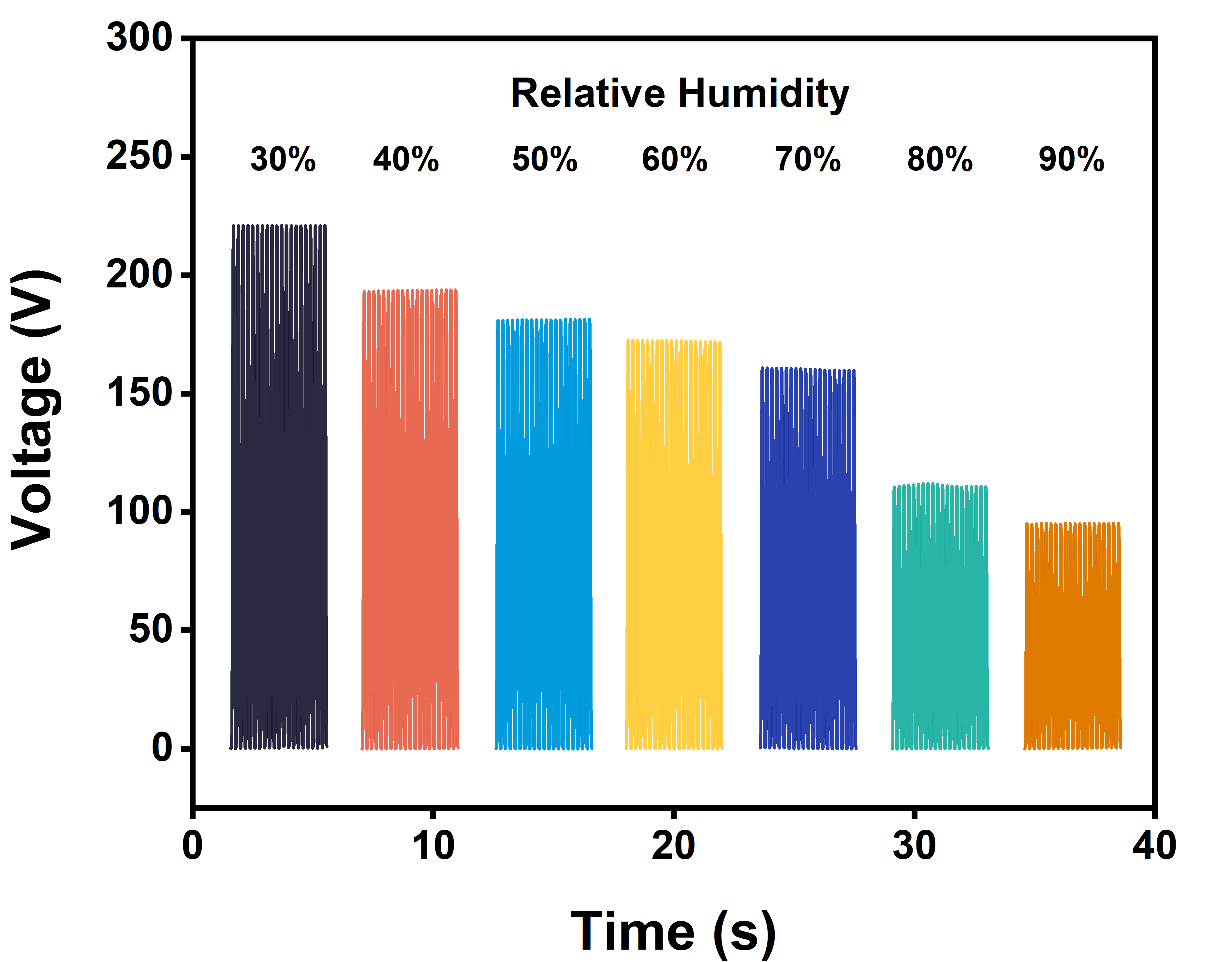


**Figure S15.** Open-circuit voltage of the TENG under different relative humidity conditions


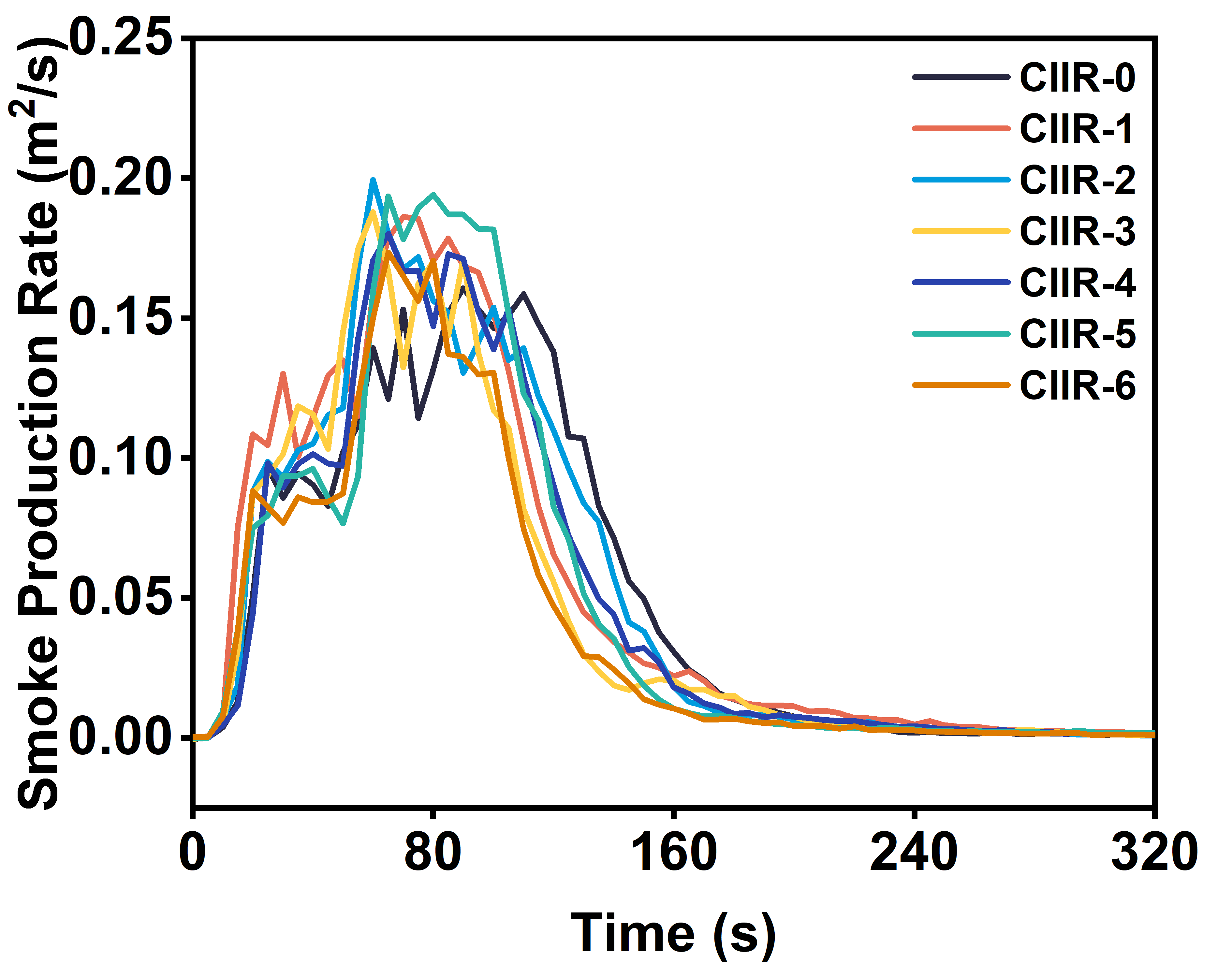


**Figure S16.** Smoke production rate during the combustion test of the control and experimental samples


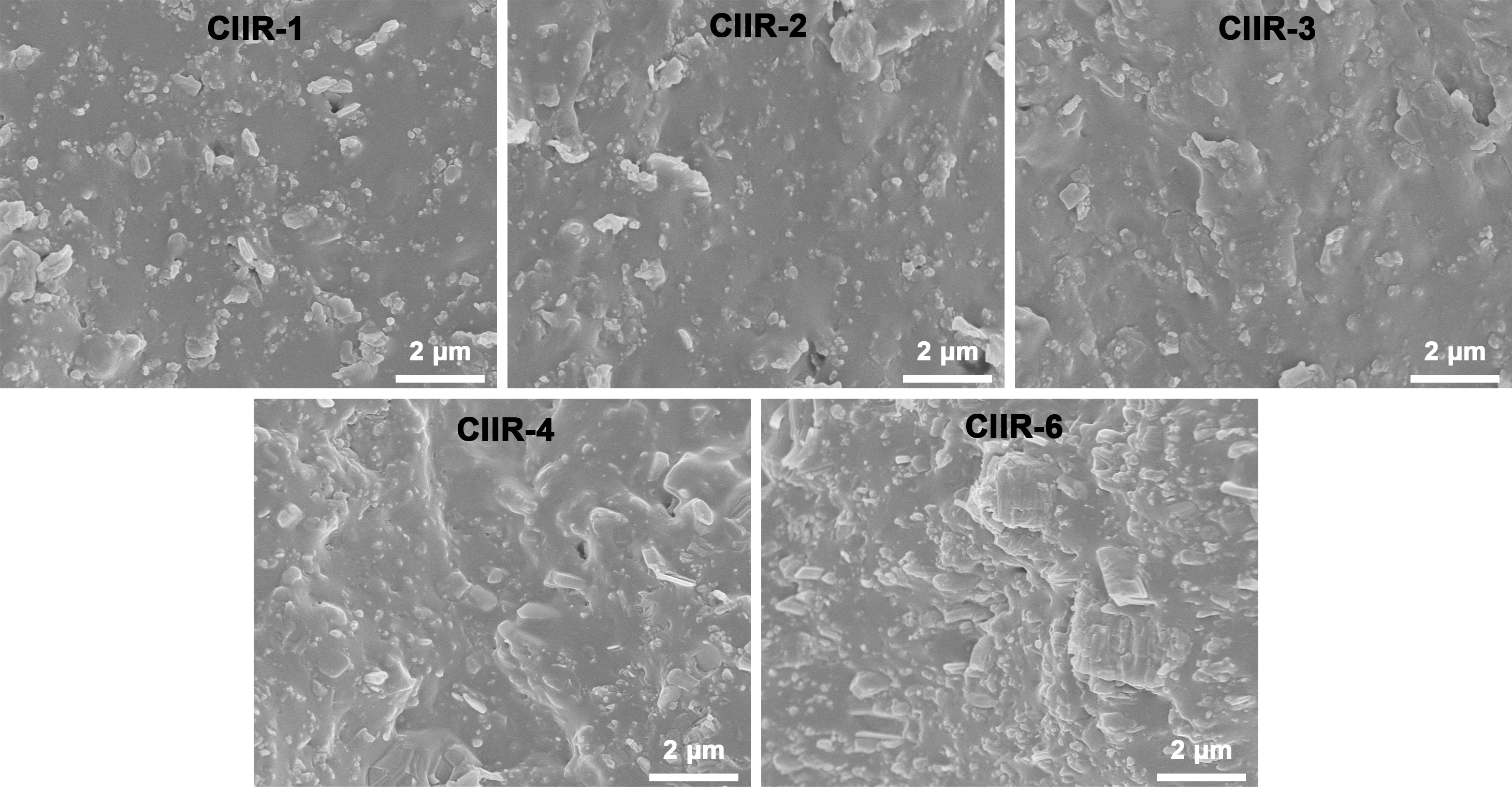


**Figure S17.** SEM images of the residual char after combustion testing of the experimental samples


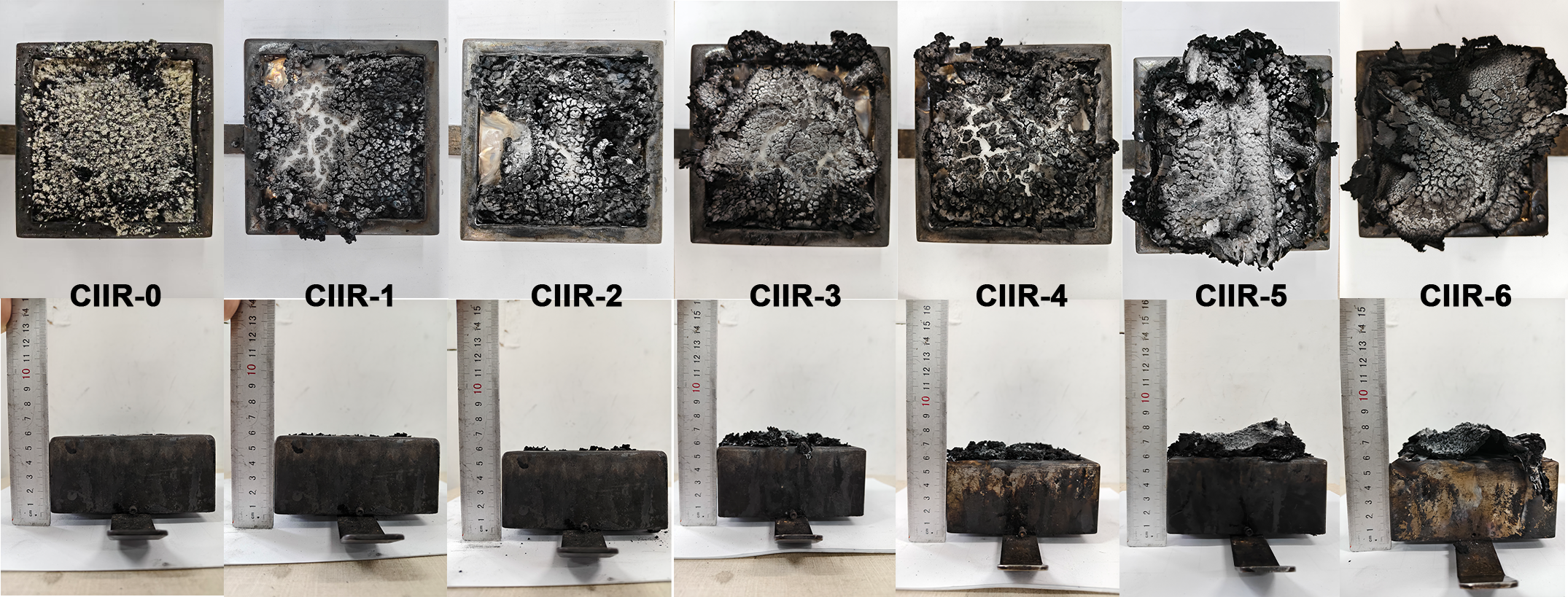


**Figure S18.** Top-view and side-view photographs of the combustion residues from control and experimental samples


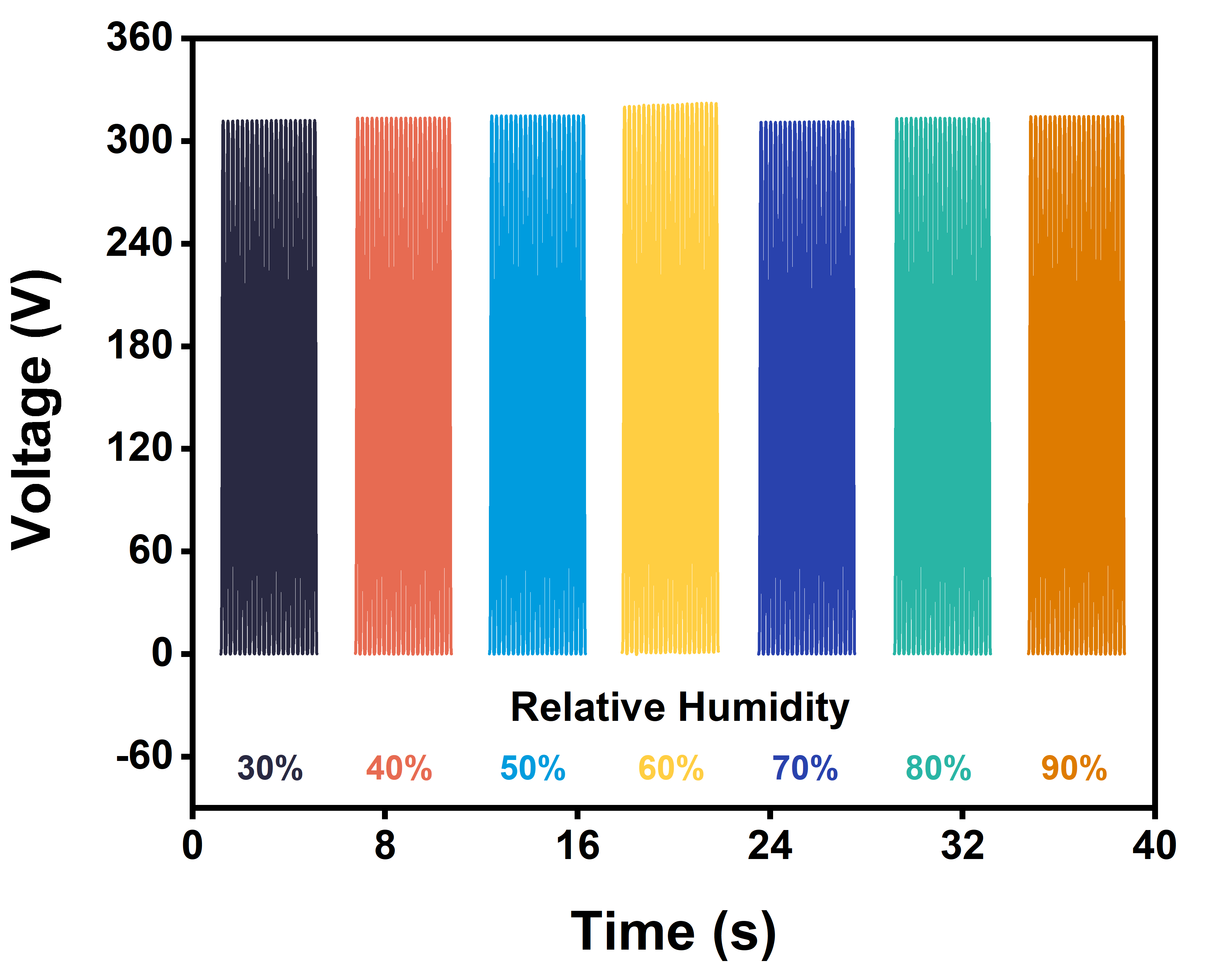


**Figure S19.** Open-circuit voltage of the encapsulated TENG under different humidity conditions


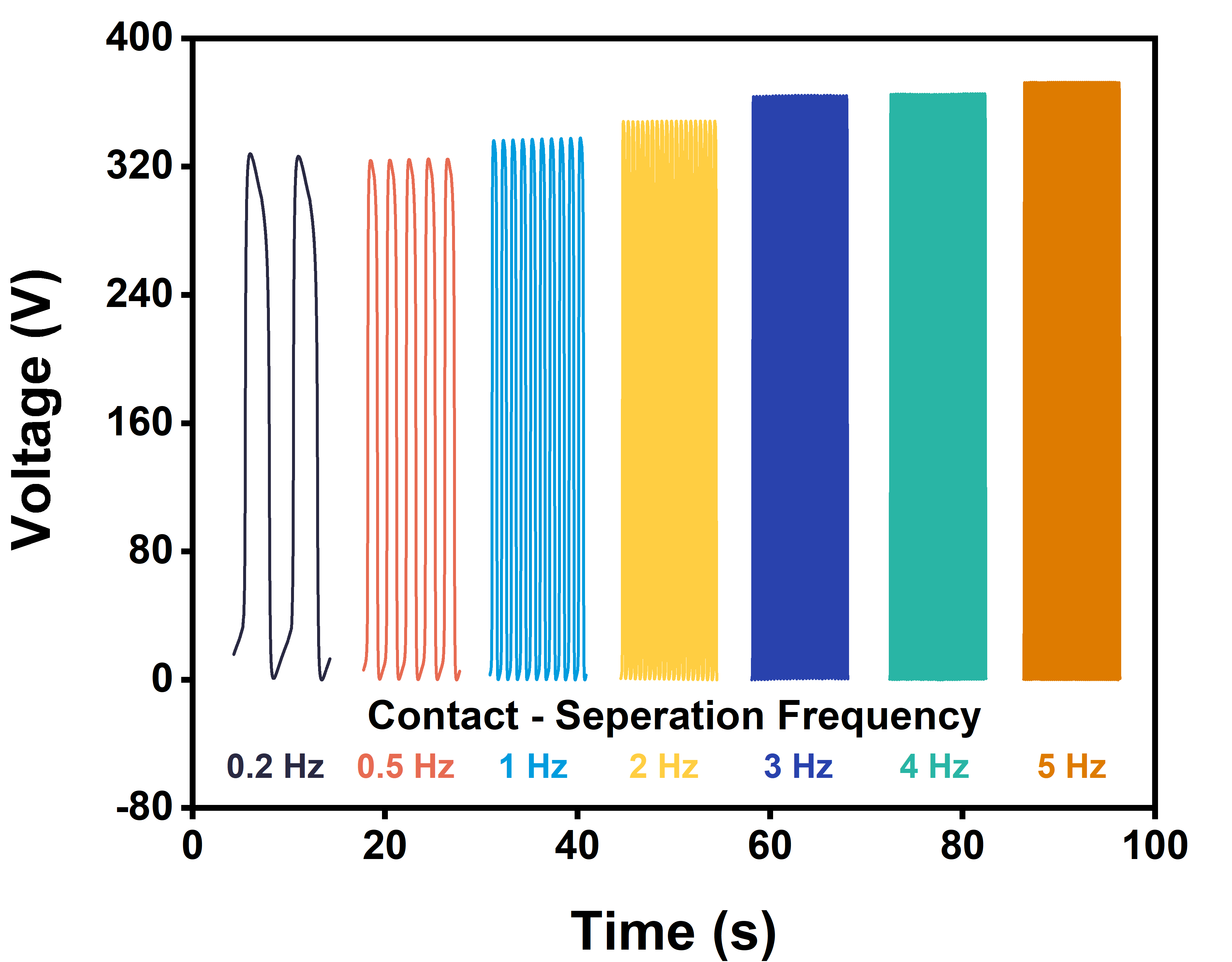


**Figure S20.** Open-circuit voltage of the encapsulated TENG under different contact-separation frequencies

**Table S1.** Formulations of CIIR-based flame-retardant and gas-barrier composites

|  |  | Sample ID | | | | | | |
| --- | --- | --- | --- | --- | --- | --- | --- | --- |
| Components | Unit | CIIR-0 | CIIR-1 | CIIR-2 | CIIR-3 | CIIR-4 | CIIR-5 | CIIR-6 |
| CIIR | g | 100 | 100 | 100 | 100 | 100 | 100 | 100 |
| Processing Aids | g | 1 | 1 | 1 | 1 | 1 | 1 | 1 |
| 4010NA | g | 2 | 2 | 2 | 2 | 2 | 2 | 2 |
| Antioxidant RD | g | 1 | 1 | 1 | 1 | 1 | 1 | 1 |
| N330 | g | 20 | 20 | 20 | 20 | 20 | 20 | 20 |
| ADP | g | - | 20 | 40 | 60 | 60 | 60 | 60 |
| MCA | g | - | 10 | 20 | 30 | 30 | 30 | 30 |
| RDP | g | - | 5 | 10 | 10 | 10 | 10 | 10 |
| Organic Clay | g | - | - | - | - | 5 | 10 | 15 |

**Table S2.** Summary of electrical performance and durability of HTV silicone rubber based TENGs

| **Ref.** | **Materials** | **Fillers** | **V_oc_** | **I_sc_** | **SCD** | **Current density** | **Durability** | **Humidity resistance** | **Flame retardancy** |
| --- | --- | --- | --- | --- | --- | --- | --- | --- | --- |
|  |  |  | V | μA | μC m^-2^ | μA cm^-2^ | Cycles |  |  |
| 53 | HTV SR-PET | 25 wt% Ag-coated glass microspheres | 200 | 4.7 | - | 0.188 | 30000 | No | No |
| 54 | HTV SR Foam | 10 wt% CNT | 91 | 2.9 | 25 | 0.182 | 2000 | No | No |
| 55 | HTV SR-Nylon | 25 wt% Ag-coated glass microspheres | 65 | 0.5 | - | 0.500 | 10000 | Yes | No |
| **This**  **work** | **HTV SR-PVDF** | **0.97 wt% 600JD carbon black** | **378** | **7.0** | **100** | **0.194** | **1000000** | **Yes** | **Yes** |

**Table S3.** List of Materials and Their Suppliers Used in This Study

| **Material** | **Specification / Notes** | **Supplier** |
| --- | --- | --- |
| Methyl vinyl silicone rubber (VMQ) | 110-2, HTV grade | Chenguang Research Institute |
| Bis(2,5-dimethyl-2,5-di-tert-butylperoxy)hexane | Trigonox 101 | Akzo Nobel |
| Hydroxyl-terminated silicone oil | — | Shenzhen Jipeng |
| TiO_2_ | Dielectric filler | Aladdin |
| BaTiO_3_ | Dielectric filler | Macklin |
| SrTiO_3_ | Dielectric filler | Aladdin |
| Fumed silica | Aerosil® 200 | Evonik |
| Carbon black N220 | Reinforcing filler | Cabot |
| Carbon black N330 | Reinforcing filler | Cabot |
| Conductive carbon black | Ketjenblack EC-600JD | Lion Corporation |
| CIIR | Chlorinated isobutylene-isoprene rubber | LANXESS |
| ADP | Aluminum diethylphosphinate | Clariant |
| MCA | Melamine cyanurate | Oceanchem |
| RDP | Resorcinol bis(diphenyl phosphate) | Oceanchem |

**Video S1.** Electric potential simulation animation of the contact-separation process between silicone rubber filled with 1 phr 600JD and PVDF

**Video S2.** Video showing the TENG lighting up over 1,900 LEDs

**Video S3.** LED logo illumination triggered by foot stepping
